# Economic and health impact of integrating smoking cessation services into NHS cancer care pathways in England: a modelling study

**DOI:** 10.1016/j.lanepe.2026.101869

**Published:** 2026-09-20

**Authors:** Maisie Green, Sam Harper, Ellen Telfer-Thomas, Sanjay Agrawal, Matthew E.J. Callister, Jenna L. Lane, Graham Warren, Rachael L. Murray, Matthew Evison, Mahdi Sheikh, Robert Malcolm

**Affiliations:** a York Health Economics Consortium, Enterprise House, Innovation Way, Heslington, York, YO10 5NQ, UK; b Lung Cancer and Thoracic Surgery Directorate, Wythenshawe Hospital, Manchester University NHS Foundation Trust, Southmoor Road, Wythenshawe, Manchester, M23 9LT, UK; c Manchester Academic Health Science Centre (MAHSC), Faculty of Biology, Medicine & Health, University of Manchester, Manchester, UK; d Institute of Lung Health, Glenfield Hospital, Groby Road, Leicester, LE3 9QP, UK; e Department of Respiratory Medicine, Leeds Teaching Hospitals, St James’s University Hospital, Beckett Street, Leeds, LS9 7TF, UK; f Leeds Institute of Health Sciences, University of Leeds, 6 Clarendon Way, Leeds, LS2 9LN, UK; g Greater Manchester Cancer Alliance, The Christie NHS Foundation Trust, Wilmslow Road, Manchester, M20 4BX, UK; h Department of Radiation Medicine, Markey Cancer Center, University of Kentucky, 800 Rose Street, Lexington, KY, 40536, USA; i School of Medicine, Applied Health Research Building, University of Nottingham, Nottingham, NG7 2RD, UK; j Early Detection, Prevention, and Infections Branch, International Agency for Research on Cancer (IARC), World Health Organization (WHO), 25 Avenue Tony Garnier, Lyon, 69007, France

**Keywords:** Smoking cessation, Cost-effectiveness analysis, Cancer survival, Quality of life, Partitioned survival model

## Abstract

**Background:**

Research has shown that smoking post-diagnosis negatively impacts cancer outcomes. This study aimed to assess the health and cost impacts of introducing smoking cessation services at cancer diagnosis for people who smoke.

**Methods:**

A cost-effectiveness model was developed using a UK National Health Service (NHS) and Personal Social Services perspective. A partitioned survival model was built and survival analysis was used to estimate the proportion of the cohort in each health state (progression-free, progressed and dead) based on smoking status. Four populations were explored: lung cancer (stage 1–3a), head and neck cancer (stage 3–4), kidney cancer, and general cancer.

**Findings:**

Over a lifetime time horizon, offering smoking cessation at diagnosis resulted in an incremental cost-effectiveness ratio of £2606, £5495 and £4055 per quality-adjusted life-year (QALY), for lung, head and neck, and general cancer, respectively. The intervention was dominant for kidney cancer. Offering smoking cessation extended life by 2–8 months and slowed cancer progression by 2–6 months.

**Interpretation:**

This analysis indicates that implementing smoking cessation into NHS cancer care is cost effective at a £20,000 per QALY willingness-to-pay threshold, and provides large health benefits. Smoking cessation aligns with the NHS 10-year plan to move from treating sickness to preventing illnesses.

**Funding:**

This study was funded by the Manchester Foundation Trust.

**Research in context**

**Evidence before this study**

Previous studies have shown that smoking during cancer treatment negatively impacts overall survival, cancer-specific mortality, and clinical outcomes, as well as increasing treatment-related complications. It has been shown that smoking cessation in people who smoked at cancer diagnosis can reduce the risk of cancer progression, cancer-specific mortality and overall mortality compared with continued smoking. An updated pragmatic search was conducted to identify newer published literature to inform the economic model to estimate the cost effectiveness of integrating smoking cessation services at cancer diagnosis in the UK. The searches were mainly focused on Google Scholar, conducted on 15th August 2025 to identify any new literature, that were not previously identified beyond 2022. Search terms for the updated pragmatic search focused on ‘smoking cessation’ or ‘tobacco use’ in combination with service areas and alternative words for ‘quit’ (abstain, stop, dehabit).

A UK study found that integrating smoking cessation services with other services, such as targeted lung health checks, is cost effective. Integrating smoking cessation services directly into the cancer care pathway for those who currently smoke was found to be cost effective in Canada^1^; however, few studies have assessed this in a UK population. Authors were previously involved in work for NICE guidelines, which produced systematic reviews, meta-analysis, and a range of economic analysis to estimate the cost-effectiveness of smoking cessation and optimising services. Search terms and details for this original comprehensive search can be identified in NICE guideline NG209.

**Added value of this study**

While the existing literature has focused on the screening phase, our paper evaluates the economic impact of smoking cessation at the specific point of cancer diagnosis. A novel cost-effectiveness model was developed to assess the impact of providing smoking cessation services alongside cancer treatment in four cancer populations (early-stage lung cancer, late-stage head and neck cancer, kidney cancer and a general cancer population). The results show that offering smoking cessation services at the point of a cancer diagnosis is cost effective, compared with the usual provision of cessation services. The results indicate longer survival per person and improved quality of life in all populations that were assessed.

**Implications of all the available evidence**

This analysis indicates that implementing smoking cessation into NHS cancer care would provide large quality of life and survival benefits, and cost a small amount of money when compared with investments in expensive cancer treatment. Implementing smoking cessation into cancer care would also contribute to the broader goals of the NHS 10-year plan. Offering smoking cessation at cancer diagnosis aligns with one of the plan’s goals to move from ‘sickness to prevention’. By reducing the number of people who smoke, first-line cancer treatments will be more effective, and fewer people will require second-line care, resulting in improvements to morbidity and mortality.

## Introduction

Tobacco smoking is a substantial preventable cause of ill-health and death worldwide.^2^ It is associated with several health problems, including cancer, cardiovascular disease, and respiratory disease.^2^ In 2023, there were an estimated 57,200 cases of cancer caused by smoking in the UK.^3^

Research shows that smoking during cancer treatment negatively impacts treatment outcomes. For example, smoking was shown to impair overall survival (OS) and cancer-specific mortality when compared with former or never smokers,^4^ and lead to worse clinical outcomes, including higher rates of disease progression.^5^ Additionally, tobacco use leads to increased treatment-related complications (such as surgical site infections and pulmonary complications following surgery) and tobacco-related comorbidities.^6^

There is growing evidence to explain the mechanisms of action between tobacco use by people with cancer and poorer cancer treatment outcomes. This includes the effects of tobacco on biological characteristics of cancer cells and altered drug metabolism, with anticancer drugs metabolised more quickly in people who smoke.^7^ Despite this, smoking rates remain high among people with cancer, including those with cancers that are strongly related to smoking (such as head and neck cancer and lung cancer).^8^

Studies have shown that smoking cessation (in people who smoked at the time of diagnosis) after cancer diagnosis can result in a reduced risk of cancer progression, cancer-specific mortality, and overall mortality compared with continued smoking.^9^, ^10^, ^11^, ^12^ In England, community stop smoking services offer tailored support to all people who smoke who are willing to quit.^13^ However, this relies on people being referred by healthcare providers or self-referral to the service.

Based on previous evidence and clinical feedback, it is expected that delivering smoking cessation services alongside cancer treatment may be more effective than traditional referral routes to standalone cessation services.^14^ Redesigning services may subsequently reduce the prevalence of smoking among people with cancer, leading to improved health outcomes and possible economic benefits. A Canadian study found that integrating smoking cessation services directly into the cancer care pathway for those who currently smoke is cost effective^1^; however, few studies have assessed this in a UK population. Previously, a UK study found that integrating smoking cessation services with other services, such as targeted lung health checks, is cost effective.^15^

This study aimed to evaluate the cost and health impacts of providing smoking cessation services alongside cancer treatment to people diagnosed with cancer who smoke. The study evaluated smoking cessation in four populations: lung cancer (stage 1–3a), head and neck cancer (stage 3–4), kidney cancer (all stages), and an exploratory general cancer population.

## Methods

### Decision problem

A cost-effectiveness model was developed, aligned with the National Institute for Health and Care Excellence (NICE) reference case and, therefore, from the perspective of the UK NHS and Personal Social Services.^16^ Outcomes were quantified in terms of quality-adjusted life years (QALYs), and both costs and QALYs were discounted at 3.5% per annum. A lifetime time horizon was implemented and a cost-effectiveness threshold of £20,000 per QALY gained was used.

The length of the time horizon was tailored to the selected population starting age. As described in the NICE manual,^16^ a lifetime time horizon was determined by the point in time when all the modelled cohort had died. Therefore, a 25-year time horizon was used for the lung cancer population, and a 30-year time horizon was implemented for the other three populations. Scenario analysis also covered shorter time horizons. Monthly cycle lengths were used to capture clinical events, costs, and impacts of cancer-related outcomes.

The model population was defined as individuals aged 18 years or older who were recently diagnosed with cancer and who currently smoke. Four populations of cancer were explored: lung cancer (stage 1–3a), head and neck cancer (stage 3–4), kidney cancer (all stages), and ‘general cancer’ (all stages). The three distinct populations were selected because the relevant cancer is linked with smoking as a cause of cancer, the available data suggested an impact on survival when quitting smoking, and to reflect a range of cancers (both early and late stage cancer).^9^, ^10^, ^11^^,^^17^ The ‘general cancer’ population reflected a mix of several different cancer diagnoses. This cancer population was an exploratory analysis because strong assumptions related to the costs and health benefits of multiple cancers had to be made. However, it is anticipated that implementation of smoking cessation services would not be limited to one cancer. Therefore, it is important to consider the potential impact on a range of different types of cancer.

### Model structure

The cohort that entered the model was split into those who quit smoking (either naturally or with the aid of smoking cessation services) and those who continued smoking. Allocation into the quit and continued groups was based on natural quit rates, plus the uptake of smoking cessation services, including the 5-year relapse rate. Within each overarching smoking status group, a partitioned survival model was built with three health states: progression-free, progressed (local recurrence of metastasis), and dead. After the initial cohort was split by smoking status, they entered the respective progression-free health state. Once individuals moved to the progressed health state, they could not move back into the progression-free health state. People in the progression-free or progressed health states may transition to the dead health state each cycle. The proportion of people that occupied each health state over a lifetime time horizon was informed by survival analysis using Kaplan–Meier (KM) estimates based on smoking status and cancer population.

The proportion of people who had quit after 1 year was used to allocate people into the quit and continued smoking groups, and people were assumed to have quit for the rest of their lives from this point onwards. 1 year of abstinence is a clinically significant marker for quitting smoking long term due to the low relapse rate.^18^^,^^19^ Therefore, 1 year is often used in clinical trials and studies as a proxy for lifelong smoking cessation, such as in the Evaluating Long-term Outcomes of NHS Stop Smoking Services study.^20^

Smoking cessation methods were available to people in both arms of the model. In the intervention arm, smoking cessation was offered at diagnosis. In the comparator arm, smoking cessation methods were available through the centralised referral or self-referral service. The differences in treatment in each arm of the model were considered, and a scenario analysis was included that assessed the impact if the treatments were the same in each arm. The value of switching to more pharmacological methods than current services was explored, based on previous work of integrating services as part of lung health checks.

In the model, everyone in the progression-free and progressed health states were at risk of smoking-related comorbidities. The smoking-related comorbidities included in the model were selected based on data availability and included: chronic obstructive pulmonary disease (COPD), coronary heart disease, myocardial infarction, type 2 diabetes, stroke, dementia, and asthma. To estimate gender-weighted risks of each comorbidity by age for people who quit or continued smoking, the first step was to estimate the prevalence of each comorbidity by age and gender for non-smokers. The method for this calculation is described in the Appendix. After the non-smoker comorbidity prevalence (exclusive of smoking impacts) were estimated, they were then multiplied with the smoking-status relative risk for the relevant comorbidity to estimate age and gender stratified comorbidity prevalences.

Fig. 1 displays the model structure, including the allocation of people into quit and continued smoking, the three model health states (progression-free, progressed and dead), and the seven comorbidities.

**Fig. 1:**
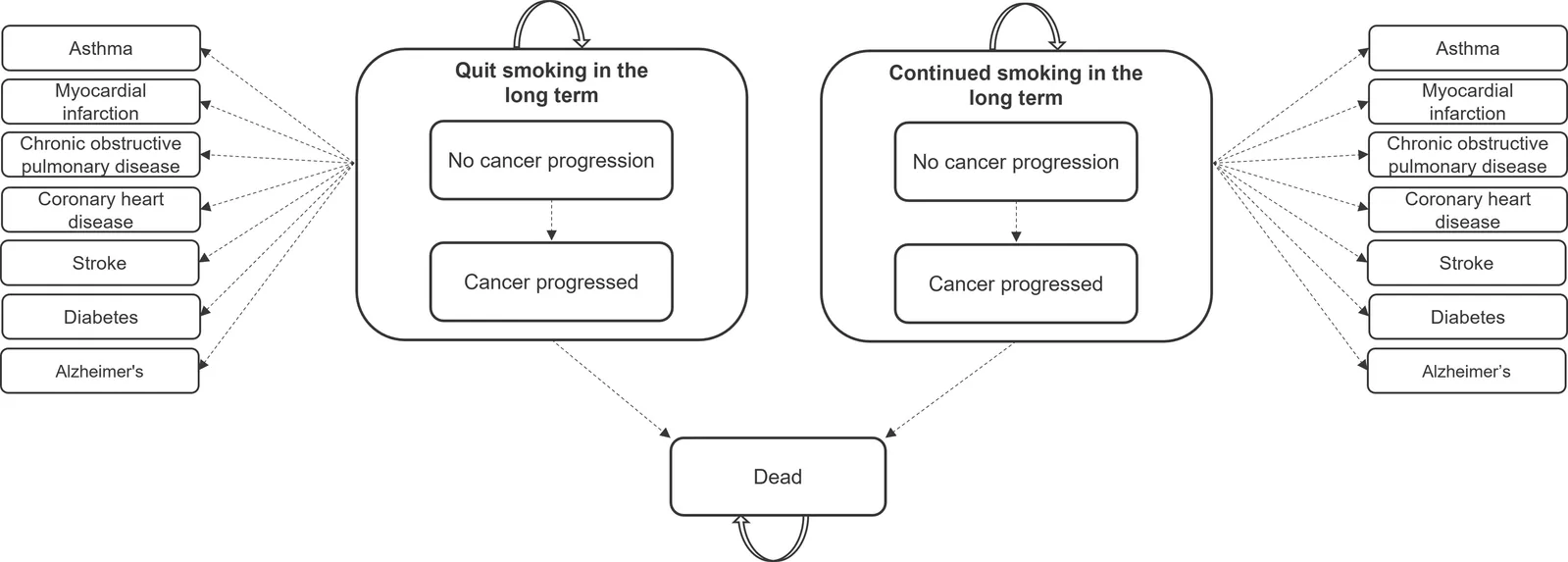
**Module structure diagram.** The arrows indicate possible cyclical movements.

### Time-to-event

People entered the model and remained in the respective ‘progression-free’ health state until they experienced cancer progression or died. Parametric survival models were used to estimate the time-to-progression and time-to-death. Hence, the parametric models were fit to predict OS and progression-free survival (PFS). To inform the time-to-event data in the model, PFS and OS KM data were required for each population for those who quit smoking and for those who continued smoking after a cancer diagnosis.

Published KM data were sourced for lung cancer, head and neck cancer, kidney cancer, and general cancer.^9^, ^10^, ^11^^,^^17^ In the head and neck and general cancer populations, KM data could not be sourced for PFS. Instead, hazard ratios were used to estimate the difference between OS and PFS from the published lung cancer KM data, where a smaller hazard ratio indicated a larger difference between OS and PFS.^9^ The hazard ratios were produced in R using the ‘survival’ package,^21^ and were 0.87 and 0.89 for quit smoking and continued smoking, respectively. This implies that for those that quit smoking, the rate of progression or death was 13% reduction in the rate of mortality.

The KM curves were uploaded to the web application “WebPlotDigitizer”, which was used to generate approximate coordinates for the KM curves.^22^ Pseudo-individual participant data (pseudo-IPD) datasets were then constructed using the extracted data with the Guyot algorithm (available in the ‘survHE’ R package).^23^^,^^24^ Once pseudo-IPD were generated, the six standard parametric models (exponential, Weibull, Gompertz, log-normal, log-logistic, and generalised gamma) were fit using the R package ‘flexsurv’,^25^ in line with the methods outlined in the NICE Decision Support Unit Technical Support Document.^26^ Regression survival analysis was then conducted in R, using the ‘survHE’ and the ‘flexsurv’ packages,^24^^,^^25^ to find the long-term extrapolations beyond the reported time periods.

From the modelled long-term extrapolations, coefficients were produced and included in the Excel economic model to calculate long-term OS and PFS for both quit and continued smoking populations in each of the four populations. It was assumed that the proportional hazard assumption would not hold between quitting smoking and continued smoking, and no treatment group covariate was included in the parametric models.

The Akaike information criterion (AIC) and Bayesian information criterion (BIC) values, along with visual graphical checks, were used to determine the best-fitting parametric model of each dataset. Clinical authors were also consulted in selecting the appropriate curves for plausibility, who confirmed that the long-term survival projections were clinically representative. However, no formal methods were followed for clinical elicitation. The chosen distributions for each dataset are presented in Supplementary Table S1 (Appendix). Survival curves are presented in Fig. 2.

**Fig. 2:**
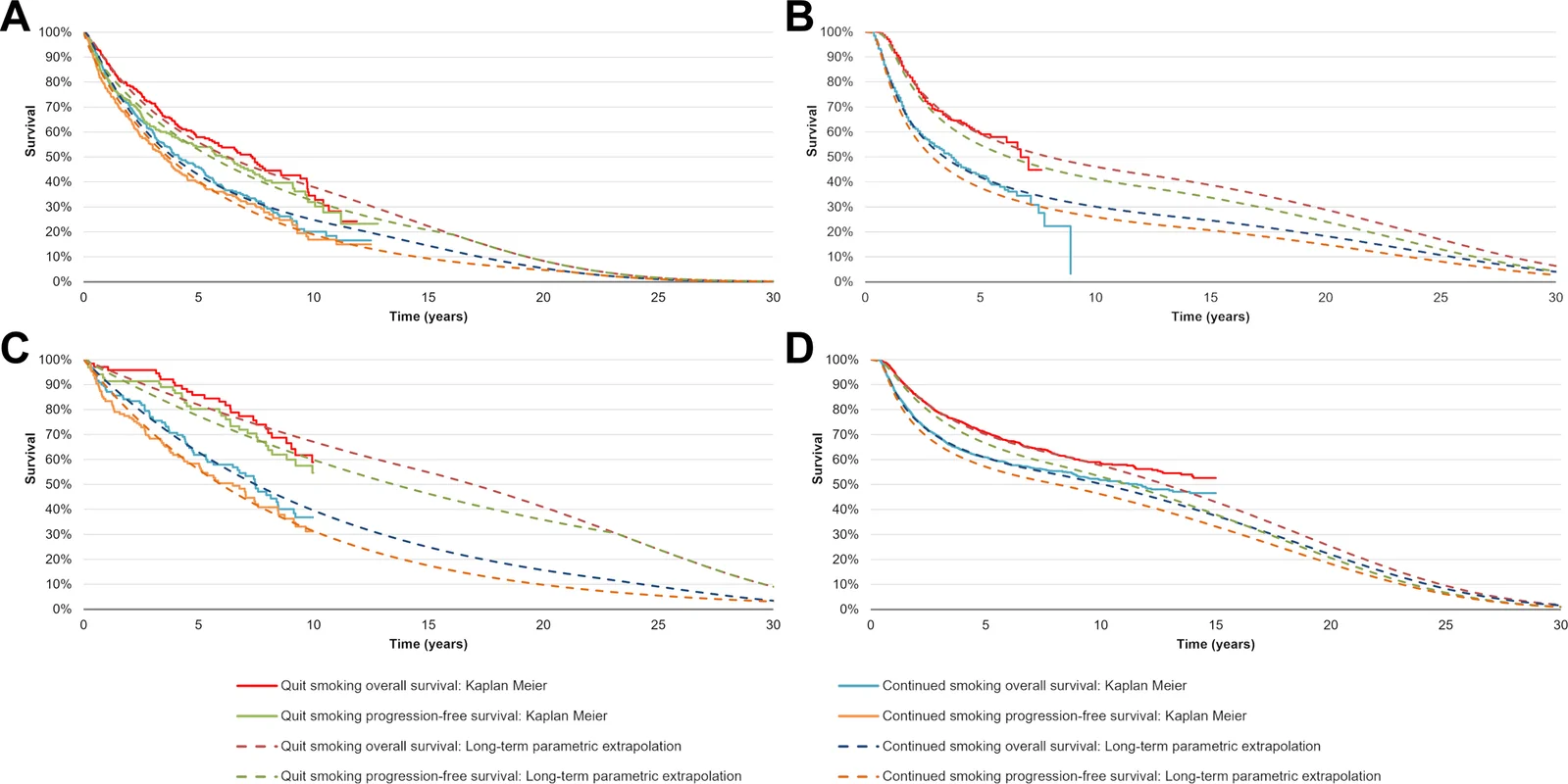
**Survival curves (long-term extrapolations and Kaplan Meier) for the lung cancer population (panel A), the head and neck cancer population (panel B), the kidney cancer population (panel C) and the general cancer population (panel D)**.

### Model parameters

The model was populated with the most appropriate available data to ensure that it was reflective of the UK population. This modelling study utilised secondary, aggregated data without direct contact with human subjects. Therefore, formal ethical approval and written informed patient consent were not required. Model parameters used to populate the model are outlined in the Appendix.

The economic model required effectiveness parameters to determine the proportion of people who quit smoking (Supplementary Table S4, Appendix). The uptake of smoking cessation when offered at diagnosis was 74.38%, sourced from the uptake of smoking cessation when offered at lung cancer screening in Murray et al. (2024).^27^ This was used as a starting proxy to understand the potential impact in cancer diagnosis, and was then varied in sensitivity analysis. The uptake for usual care was 17.38%, sourced from Wu et al. (2018).^28^ The proportion of people receiving different smoking cessation methods when offered at lung cancer screening was sourced from Murray et al. (2024),^27^ and for standard of care, was sourced from Williams et al. (2023).^14^

The probability of quitting smoking at six months, including the natural quit rate and the proportion quitting with behavioural support, is detailed in Supplementary Table S5 (Appendix). The natural quit rate probability was assumed to be 5% based on the average quit rate for the control group in Livingstone-Banks et al. (2019).^29^ The proportion quitting with behavioural support was sourced from NICE NG209.^30^ Furthermore, the relative rate of quitting at 6 months for alternative smoking cessation interventions was sourced from Lindson et al. (2023) and NICE NG209.^30^^,^^31^ The relapse rate when using smoking cessation between 6 months and 5 years was sourced from NICE NG209 and Hawkins et al. (2010).^30^^,^^32^ As these data reflected a general population, scenario analysis was conducted to investigate the impact of the potential for increased quitting rates specifically in lung cancer.^33^

The relative risk of comorbidities for current smokers and former smokers is presented in Supplementary Table S8 (Appendix), and was sourced from the Royal College of Physicians (2018) and Prescott et al. (1998).^34^^,^^35^ Where relative risk differed by gender, values were stratified accordingly. The cost and utility decrement for each comorbidity are also detailed in Supplementary Table S9 (Appendix). Annual costs were calculated and, where necessary, were inflated using the Personal Social Services Research Unit (PSSRU) 2024 inflation indices.^36^ Prevalence inputs for smoking and for each comorbidity are detailed in Supplementary Tables S10–S18 (Appendix).

The unit costs of smoking cessation interventions are detailed in Supplementary Table S19 (Appendix). The costs were for the year 2025, with any dated costs inflated using the PSSRU 2024 inflation indices.^36^

It was assumed that there would be a digital cost associated with the inclusion of smoking cessation services at cancer diagnosis as part of NHS care. However, this cost is currently unknown and is difficult to determine. Therefore, to account for the cost of restructuring NHS systems, the cost of an e-consultation (£45.98) was used as a proxy, sourced from Jones et al. (2024).^36^

Treatment costs for cancer progression were calculated using pack and treatment unit inputs, including the pack cost, unit size, pack size, dose, cycle length, and duration. Pack information was sourced from the British National Formulary and the drugs and pharmaceutical electronic market information tool.^37^^,^^38^ Treatment information was sourced from NICE technology appraisal documents. See the Appendix for further costing details.

The monthly progression-free and progressed health state resources were sourced from NICE technology appraisal documents, details of which can be found in Supplementary Table S26, S31, and S36 (Appendix). These inputs were applied to the unit costs associated with each resource, sourced from the National Cost Collection.^39^ A one-off, end-of-life cost was applied to the modelled cohort upon entering the ‘dead’ health state. A cost of £13,409 was sourced from the PSSRU, using the hospital and social care cost of dying from cancer.^36^

Health state utilities for each population are detailed in Supplementary Table S39 (Appendix). Utilities were applied multiplicatively to background utilities, using the age and proportion male values. The background utility was informed by EQ-5D data published by the NICE Decision Support Unit.^40^

Finally, the survival analysis was adjusted in the cost-effectiveness model to ensure that the instantaneous hazard of death for those with cancer (smoking or not smoking) could never be lower than the general population mortality rates for the relative age of the modelled cohort in the UK. Similarly, the proportion in the progression-free health state were capped in the model by the proportion alive. The annual general population mortality rates were adjusted by the gender distribution reported by the Office for National Statistics.^41^

### Model assumptions

Due to paucity of published data, assumptions were made around model inputs and structural elements (Table 1).

**Table 1:** Key modelling assumptions.

Assumption	Rationale
For the general cancer population, it was assumed that there would be equal progression risk between long-term smoking quitters and current smokers or people who relapse.	This assumption was due to a paucity of evidence to populate progression risks. However, this approach is a more conservative approach. Setting progression rates to be equal between smoking outcome arms avoided introducing unnecessary uncertainty into the model.
Due to a lack of available data, the overall and progression-free survival data for both kidney and lung cancer were sourced from a Russian population. It was assumed that this data is applicable to the UK setting. In the head and neck cancer population, data in a UK and Latin American population were included for risk estimates of quitting smoking and overall survival.	This assumption is supported by clinical judgement. Clinicians confirmed that there is no genetic or population characteristics that would lead to substantial differences in outcomes between a Russian population and UK population. Both UK and Latin American populations provided similar risk estimates for quitting smoking and overall survival. Therefore, it was assumed that there were not substantial differences in these estimates.
The National Disease Registration Service data that was used for lung cancer did not split out stage 3a from stage 3b. Therefore, the data used for ‘stage 3a’ included some people with stage 3b cancer. Therefore, the proportion of people with stage 3a cancer was expected to be an overestimation.	The assumption enabled the use of robust data from the National Disease Registration Service. The subsequent potential of overestimating stage 3a inputs was a noted limitation. As this approach was used in both arms of the model, it did not likely contribute to an incremental difference between arms.
It was assumed that the effectiveness of smoking cessation in reducing the proportion of the population that smoked was the same in a cancer population as it is in the general population.	This was a conservative assumption given that the effectiveness of smoking cessation is likely greater in a cancer population. This assumption was also necessary due to a lack of data among people with cancer. The impact of a relatively higher rate of quitting for those with lung cancer was investigated.
It was assumed that everyone diagnosed with cancer would receive first line treatment and therefore only second line treatment associated with progression was costed for within the model.	These conservative assumptions reflect a pragmatic approach to facilitate modelling of complex cancer care pathways.
It was assumed that treatments for cancer were not dependent on smoking status.	
It was assumed that the relapse rate of people beyond the first year of smoking cessation was equal to that in subsequent years.	This is a conservative assumption as relapse rates are expected to decline after the first year of smoking cessation.^32^ Using this conservative approach avoids overestimating rates of continued smoking cessation which may bias the model in favour of the intervention.
To calculate the number of people who moved from progression-free to progressed, it was assumed that of the people moving out of the progression-free health state, the proportion who died (rather than progressed) was equal to the hazard rate for that model cycle. This assumed that the rate of death is consistent between progression-free and progressed.	This was a simplifying assumption due to a lack of access to individual participant data from the sources used in the survival analysis estimates. This is expected to be a conservative assumption, as the rate of death is likely higher in a progressed population that a progression-free.
The QALY decrement for dementia used a proxy-rated utility value.	Self-rated dementia utilities are considered irreflective of the disease as they are often the same as or higher than the general population utility.
Head and neck cancer data was collected before different staging rules were applied to HPV-related cancers. This may mean that under current definitions, some people in the late-stage head and neck cancer populations, are no longer considered late stage.	This is a limitation of the available data and should be noted when considering the survival in this population. However, it was not possible to stratify the data to isolate those with HPV-related cancers.
The risk and subsequent resource-use, cost and HRQoL impact of comorbidities was not dependent on the cancer diagnosis, nor the level of cancer progression.	This was a simplifying assumption due to a lack of data with which to inform the interaction between quitting and the risk of specific comorbidities. It is anticipated that this is a conservative assumption, as the risk of certain smoking-related comorbidities when in combination with cancer could be higher than the general population.

HRQoL, health-related quality of life; QALY, quality-adjusted life year.

### Role of the funding source

This study was funded by the Manchester Foundation Trust. The study sponsor was involved in the conceptualisation of the study, reviewing results of the analysis, reviewing the manuscript, and the decision to submit the paper for publication.

## Results

### Deterministic base case results

A summary of the base case results comparing smoking cessation offered at cancer diagnosis with usual care for the four populations is displayed in Table 2. Outcomes are relative to the cost-effectiveness threshold of £20,000 per QALY gained and presented on a per-person basis. It is estimated that offering smoking cessation at diagnosis is cost effective over a lifetime time horizon for all four populations.

**Table 2:** Summary of per-person cost and health impacts of the smoking cessation intervention at diagnosis for all four populations.

	Smoking cessation intervention at diagnosis	Usual care	Incremental
Lung cancer (stage 1–3a) results			
Median time to death	4.25 years	4.08 years	0.17 years
Median time to event	4.00 years	3.83 years	0.17 years
Cost per person	£61,329	£61,037	£292
QALYs per person	5.65	5.48	0.17
Life years per person	3.19	3.07	0.11
ICER			£2606
Incremental NMB			£1951
Incremental NHB			0.10
Head and neck cancer (stage 3 and 4) results			
Median time to death	4.00 years	3.67 years	0.33 years
Median time to event	3.42 years	3.08 years	0.33 years
Cost per person	£40,064	£38,990	£1074
QALYs per person	4.13	3.93	0.20
Life years per person	6.80	6.51	0.29
ICER			£5495
Incremental NMB			£2834
Incremental NHB			0.14
Kidney cancer (all stages) results			
Median time to death	8.58 years	7.92 years	0.67 years
Median time to event	6.83 years	6.33 years	0.50 years
Cost per person	£39,517	£39,707	−£189
QALYs per person	4.52	4.26	0.26
Life years per person	8.40	7.96	0.45
ICER			Dominant
Incremental NMB			£5362
Incremental NHB			0.27
General cancer (all stages) results			
Median time to death	10.75 years	10.42 years	0.33 years
Median time to event	8.92 years	8.42 years	0.50 years
Cost per person	£58,716	£58,324	£392
QALYs per person	5.06	4.96	0.10
Life years per person	8.60	8.47	0.13
ICER			£4055
Incremental NMB			£1543
Incremental NHB			0.08

ICER, incremental cost-effectiveness ratio; NHB, net health benefit; NMB, net monetary benefit; QALY, quality-adjusted life year.

The per-person breakdown of costs for each population is provided in the Appendix (Supplementary Tables S42–S45). Treating lung cancer, head and neck cancer and general cancer was shown to cost an additional £248, £996, and £398, respectively. This is a result of the progression-free cost being substantially higher in the intervention arm compared with usual care. The increased cost of treating progression-free cancer is due to the decrease in people moving to the progressed health state, or dead, in the intervention arm. Therefore, more people remain in the progression-free health state, and a higher cost is incurred due to post-treatment impacts on health.

Kidney cancer was the only population where treating cancer was cheaper in the intervention arm compared with usual care (by £310). Treating people in the progression-free health state was more costly in the intervention arm. However, this was offset by the cost savings for second-line treatment (incurred when people progress) and end-of-life care (incurred when people die).

For all populations, offering smoking cessation at diagnosis increases the median time to death and the median time to cancer progression. The estimates indicate that of the costs that the NHS incurs during the course of cancer treatment, the proportional contribution of smoking cessation at the point of diagnosis ranges from 0.34% to 0.53%, excluding upfront first-line treatments and third-line and onwards treatment costs (Supplementary Table S46, Appendix).

### Deterministic sensitivity analysis

The univariate impact for a range of parameters was tested in deterministic sensitivity analysis (DSA). When all parameters are varied independently, the main drivers of the incremental net monetary benefit (NMB) for all populations are: cohort starting age, proportion quitting with behavioural support, uptake of smoking cessation when offered at diagnosis, utility value for progression-free cancer, and utility value for progressed cancer (Supplementary Figs. S1–S4, Appendix).

### Probabilistic sensitivity analysis

Probabilistic sensitivity analysis (PSA) was conducted to quantify the level of confidence in the results, relating to uncertainty in the model inputs. Using a willingness-to-pay threshold of £20,000 per QALY gained, implementing smoking cessation services during lung cancer diagnosis was cost effective in 99.70% of 1000 iterations, and was dominant in 30.00% of iterations. In the head and neck cancer population, smoking cessation was cost effective in 99.90% of the 1000 iterations. In the kidney cancer population, smoking cessation was cost effective in 100.00% of the 1000 iterations and was dominant in 70.40% of iterations. Finally, in the general cancer population, smoking cessation was cost effective in 97.80% of the 1000 iterations.

The cost-effectiveness planes are presented in Fig. 3, and further detail is contained in the Appendix (Supplementary Tables S47–S50).

**Fig. 3:**
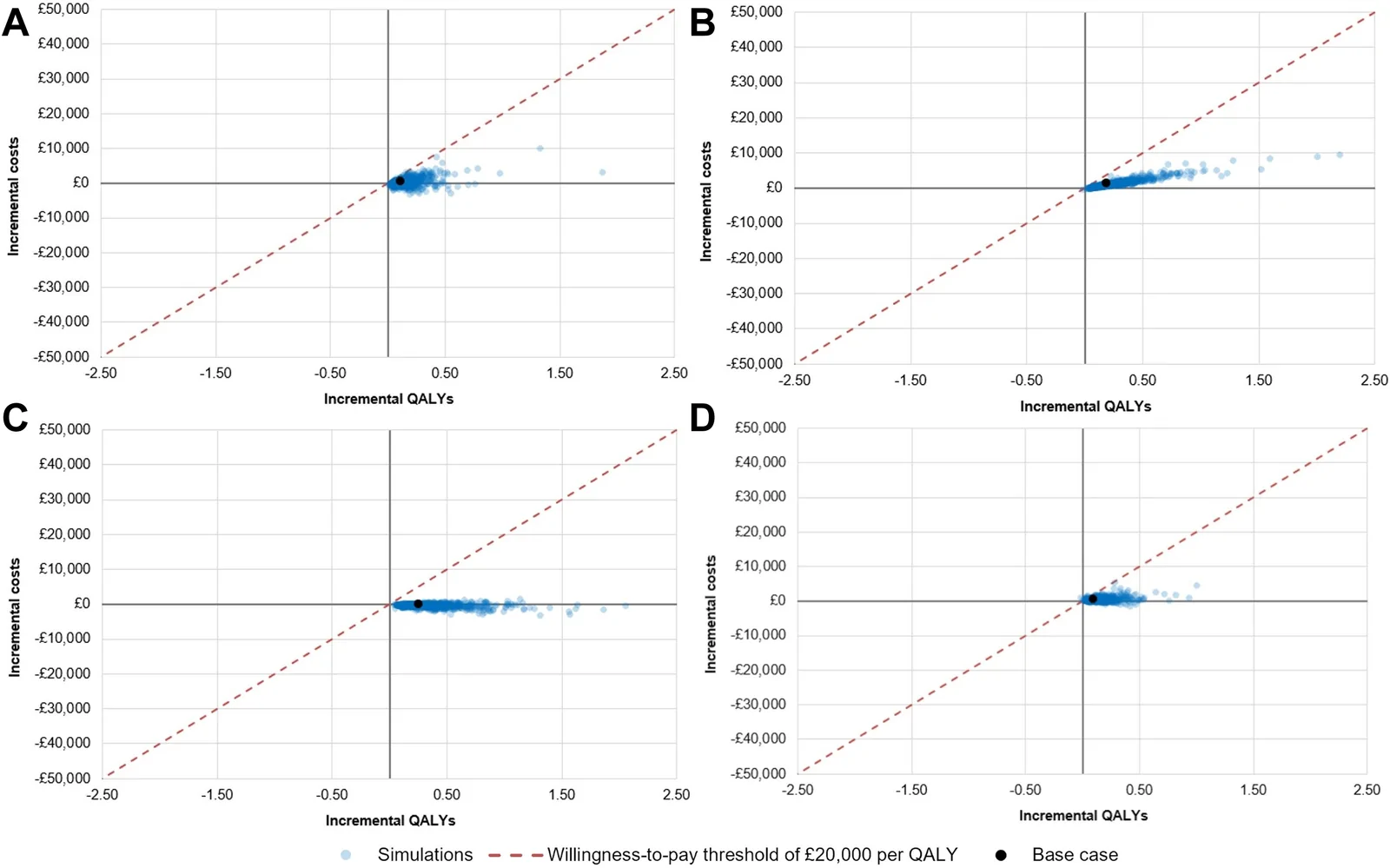
**Cost-effectiveness planes for the lung cancer population (panel A), the head and neck cancer population (panel B), the kidney cancer population (panel C) and the general cancer population (panel D).** Abbreviations: QALY, quality-adjusted life year.

### Threshold analysis

Threshold analysis was conducted to determine the range of results generated when the uptake of smoking cessation and the cost of an e-consultation (to reflect the cost of implementing smoking cessation) were varied (with all other parameters held constant). The parameter value where smoking cessation services are no longer cost effective relative to a £20,000 per QALY gained threshold was also determined. The crossing of this threshold is indicated by the incremental NMB dropping below £0.

The results indicated that the ICER would fall below £20,000 per QALY gained when uptake of smoking cessation is between 12% and 14% (depending on the population). If the uptake of smoking cessation at cancer diagnosis is higher than usual care, the intervention is likely to be cost effective. Higher uptake results in greater benefits. Further, the intervention is no longer cost effective when the cost of implementing smoking cessation services is between £2000 and £6600 (depending on the population).

The degree of change in the incremental NMB is displayed in Supplementary Fig. S5–S12 (Appendix).

### Scenario analysis

A range of scenario analyses were conducted, including scenarios investigating the impact of different parametric models, time horizon lengths, and equal treatments being given across pathways. Table 3 displays per-person results for different time horizons. Results of the other scenario analyses are presented in the Appendix. The results of each scenario analysis conducted align with the overall narrative of the results: that smoking cessation services at cancer diagnosis is a cost-effective approach.

**Table 3:** Scenario analysis per-person results of the smoking cessation intervention at diagnosis for all four populations across different time horizons.

Time horizon	Per person results	Population			
		Lung cancer	Head and neck cancer	Kidney cancer	General cancer
1 year	Incremental cost	−£124	−£124	−£158	−£128
	Incremental QALY	0.00	0.01	0.00	0.00
	**ICER**	**Dominant**	**Dominant**	**Dominant**	**Dominant**
5 years	Incremental cost	−£350	£99	−£760	−£89
	Incremental QALY	0.03	0.06	0.06	0.04
	**ICER**	**Dominant**	**£1626**	**Dominant**	**Dominant**
10 years	Incremental cost	−£157	£431	−£940	£94
	Incremental QALY	0.7	0.11	0.16	0.08
	**ICER**	**Dominant**	**£3810**	**Dominant**	**£1471**
15 years	Incremental cost	£38	£692	−£847	£226
	Incremental QALY	0.10	0.15	0.28	0.11
	**ICER**	**£388**	**£4554**	**Dominant**	**£2742**
Lifetime (base case)	Incremental cost	£292	£1074	−£189	£392
	Incremental QALY	0.11	0.20	0.45	0.13
	**ICER**	**£2606**	**£5495**	**Dominant**	**£4055**

ICER, incremental cost-effectiveness ratio; QALY, quality-adjusted life year.

## Discussion

The intention of this study was to estimate the cost-effectiveness of introducing smoking cessation services at the point of cancer diagnosis across four different populations compared with usual care. The results indicate that offering smoking cessation services at the point of a cancer diagnosis is cost effective over a lifetime time horizon, compared with usual cessation services (centralised referral or self-referral). The incremental NMB is £1951, £2834, £5362 and £1543 for lung, head and neck, kidney, and general cancer, respectively. It is estimated that around 57,000 people are diagnosed with cancer due to smoking each year.^3^ Therefore, applied to the whole population, the potential NMB could be upwards of £88 m (general cancer population). However, further research is required to estimate the population-level impact.

The results indicate longer survival and improved quality of life in all populations when smoking cessation is offered. The per-person cost (including the costs of cancer treatment, comorbidity treatment, and smoking cessation) ranges from a £189 saving in the kidney cancer population to an increase of £292, £1074 and £392 in the lung, head and neck, and general cancer populations, respectively. PSA results demonstrate consistency with the deterministic results, and that offering smoking cessation at cancer diagnosis is cost effective in 99.70% (lung cancer), 99.90% (head and neck cancer), 100.00% (kidney cancer), and 97.80% (general cancer) of the iterations at a £20,000 per QALY gained threshold. At the time of writing, NICE were planning to increase its cost-effectiveness threshold. This change does not impact the direction of results. The increase in costs within some populations is driven by people living longer post-cancer treatment, where they are likely to require further healthcare resource use. However, in shorter time horizons, the redesign of services is likely to be cost saving.

The implementation of smoking cessation at cancer diagnosis was assumed to be similar in terms of costs and uptake to when it was introduced at lung cancer screening. However, this may differ by cancer and by node of diagnosis (i.e., incidentally diagnosed). Threshold analysis reveals that offering smoking cessation at the point of cancer diagnosis is no longer cost effective when the uptake of smoking cessation is between 12% and 14% (population dependent). This is substantially lower than current uptake estimates where similar policies have been adopted. For example, in a US study, 28.3% of patients with cancer who smoked received at least one type of cessation treatment.^42^ Further, a UK study found that the uptake of smoking cessation services when offered during lung cancer screening is 74.84%,^27^ because the behaviour change associated with making services easier to access encourages greater uptake. Threshold analysis also demonstrated that the intervention is no longer cost effective when the cost of implementing changes to smoking cessation services is between £2000 and £6600 (depending on the population). Therefore, ensuring that uptake rates are above 15% and per-person intervention costs are less than £2000 will lead to a cost-effective outcome in this analysis.

The results indicate that while offering smoking cessation services at cancer diagnosis increased per-person costs in some populations, the cost is small compared with that of smoking-related diseases. For example, the estimated average annual cost per person with coronary heart disease is £6648 (inflated to a 2023/24 cost year from 2018 costs^43^). Previous literature estimated the cost savings associated with quitting smoking, and the consensus is that, due to data biases and unquantified benefits, they are likely a conservative estimate of the impact on costs.^30^^,^^44^, ^45^, ^46^ Therefore, long-term costs may not increase, or not by the amount estimated in the model. This is because evidence suggests that smoking increases the risk of many healthcare conditions, beyond just the seven comorbidities included.

The results in Supplementary Table S46 (Appendix) demonstrate that the costs from smoking cessation services contribute a small proportion (0.34% to 0.53%) of the estimated total costs the NHS incurs of treating cancer per person. Additionally, there is a large proportional cost difference between offering smoking cessation services and many of the cancer treatments recommended. For example, NICE recommended the use of durvalumab for treating limited-stage small cell lung cancer,^47^ and nivolumab for treating head and neck cancer in adults.^48^ For durvalumab and nivolumab, the per-person cost of treatment was estimated to be £141,335 and £31,693, respectively (Supplementary Tables S42–S45, Appendix). The two drugs demonstrated cost effectiveness, albeit at a substantially greater risk to the healthcare system if they do not deliver on the clinical benefits that ensure their cost effectiveness, due to high per-person treatment costs. Offering smoking cessation may cost the NHS money in the long term, but it would be associated with a lower risk than the cancer treatments due to its low per-person cost (£257), which includes an e-consultation and the smoking cessation treatments.

The novelty of the analysis is not that smoking cessation is cost-effective; it is that the careful design and integration of services can be very important to achieve good value for money. Implementing smoking cessation into cancer care is an approach that would contribute to the broader goals of the NHS 10-year plan.^49^ Offering smoking cessation at cancer diagnosis aligns with one of the plan’s goals to move from ‘sickness to prevention’. By reducing the number of people who smoke, first-line cancer treatments will be more effective, and fewer people will require second-line care, resulting in improved morbidity and mortality. This is demonstrated by the estimated improvement in median time-to-progression and time-to-death when smoking cessation services are introduced at cancer diagnosis. Hence, implementing smoking cessation into cancer care is a preventative measure that improves cancer outcomes and reduces costs for treating both early-stage and late-stage cancer.

Supporting literature indicates that integrating smoking cessation services with other healthcare services, such as lung health checks, increases the proportion of people who successfully stop smoking.^50^^,^^51^ However, it is important to note that these healthcare services are different populations, so the effect may not be perfectly transferable. For instance, in a healthier population, uptake of services may differ compared with a population who are sicker.

Importantly, smoking can be linked to a wide range of impacts beyond what is captured in the model. The co-morbidities captured within the model for smoking are based on the strongest evidence of links to smoking. However, past reports have linked smoking to a much wider range of comorbidities. Therefore, it is likely that the benefits of stopping smoking are a more conservative estimate of the benefit, given that there are likely a range of other impacts not quantified in this analysis. Additionally, higher cessation rates would reduce second-hand smoke exposure for people who spend time with those who have stopped smoking. Second-hand smoke exposure is associated with a range of conditions, including ischaemic heart disease, COPD, lower respiratory tract infections, intracerebral haemorrhage, and ischaemic strokes.^52^ Reducing this value by improving the efficacy of smoking cessation services, therefore, provides an opportunity for the NHS to reduce the substantial morbidity, mortality, and economic burden associated with second-hand smoke exposure.^53^

Another important consideration is the impact that the change in smoking cessation services may have on health inequalities. For all cancers combined in the UK, mortality rates are almost 60% higher in people living in the most deprived areas compared with the least.^54^ Furthermore, tobacco smoking is the largest cause of health inequalities in England.^55^ Therefore, interventions that improve the efficacy of smoking cessation have the potential to reduce health inequalities.^56^ Love-Koh et al. (2020) conducted a distributional cost-effectiveness analysis to consider the population health and health inequality impacts of several smoking cessation interventions.^57^ This study found that all smoking cessation interventions assessed reduced the absolute health inequality. Consequently, implementing smoking cessation into cancer care will likely reduce health inequalities alongside the benefits detailed in this analysis.

Finally, it is important to consider capacity within the system to deliver smoking cessation services. Integrating services is likely to be a good spend of money but is likely to require initial investment to cover additional staff and resources to deliver these services on a wider scale. Equally, a more targeted approach to existing services could be taken, in this case shifting resources to specific target areas, from the centralised services. The impact of this would be increasing wait times for those outside of targeted areas, not accessing within integrating services. Rollout of any policies should consider capacity alongside the economic benefits and value for money.

As with all economic models, assumptions were necessary where data were limited. Firstly, the OS and PFS data for both lung and kidney cancer were sourced from a Russian population, and it was assumed that this data applies to the UK setting. Consulted oncologists confirmed that there are no genetic or population characteristics that would lead to differences in outcomes between the Russian and UK populations, so this assumption was deemed reasonable.

Other simplifying assumptions were necessary. It was agreed that the lung cancer population would be limited to stage 1 to 3a because the treatment of stage 3b and 4 varies substantially. The National Disease Registration Service data were used to determine the stage split for the lung cancer population. However, this data does not split out stage 3a from stage 3b. Therefore, the proportion of people with stage 3a cancer is likely an overestimation. These proportions were used in the model to determine the progression treatment cost for lung cancer and was varied in the sensitivity analysis to mitigate this limitation.

The impact of alternative treatments for those who quit smoking (versus those who continued) was not reflected in this analysis, albeit may have a substantial impact that favours the cost-effectiveness of introducing smoking cessation experts at the point of cancer diagnosis. Given surgery is more likely to be indicated for people with lung cancer who quit smoking,^58^ this is particularly relevant for a lung cancer population. For those that progress from early stage cancer lung cancer to late-stage, recent changes in immunotherapies may extend survival greater than the curves used. Equally, this is also the case for kidney cancer and head and neck cancer, where the treatments may differ by smoking status, impacting the underlying survival data.

However, this is expected to have minimal impact on the results, based on the proportion that progress, the relative impact of smoking, and the fact that newer treatments are more expensive, which will counteract the health impact, with respect to economic outcomes. Taking all of these factors into account, it is unlikely to change the direction of results for early stage lung cancer.

A further limitation related to data availability is the difference in the population characteristics between the participants in the studies that report KM curves and the wider literature on cancer. For instance, the proportion of the participants in the KM studies that are male is approximately 80.00% for all three cancer populations (lung, head and neck, and kidney cancer).^9^^,^^10^^,^^59^ Comparatively, the proportion of the population with these cancers that are male is around 50%–70%.^60^, ^61^, ^62^ Consequently, the survival estimates used in the model are potentially an inaccurate representation of cancer survival in the general population. Sensitivity analysis was conducted to vary these parameters.

Another limitation is that the natural quit rate probability and the relapse rate when using smoking cessation were sourced from non-cancer populations. This was due to a lack of data among patients with cancer. The natural quit rate was assumed to be 5% based on the average quit rate for the control group in Livingstone-Banks et al. (2019).^29^ However, the natural quit rate is the same in both arms, so incrementally, there will only be a small difference, as indicated by the scenario analysis (Supplementary Table S58, Appendix). The relapse rate when using smoking cessation between 6 months and 5 years was sourced from NICE NG209 and Hawkins et al. (2010).^30^^,^^32^ However, the quit rate and relapse rate may differ in cancer patients.

Finally, there are limitations related to the general cancer population, which is an exploratory analysis whereby multiple cancer sites are considered. Given that it is not possible to determine a treatment cost or utility value to represent all cancers, an average of the lung cancer and head and neck cancer costs and utilities was employed. In the study where the KM curve is sourced, breast cancer (n = 790), haematologic cancer (n = 375), and genitourinary cancer (n = 363), along with lung cancer (n = 782) and head and neck cancer (n = 587), are the five most common reported cancers.^11^ The economic outcomes for this population should, therefore, be treated with caution, but are still useful for considering wider rollout of the policy.

### Conclusion

This analysis provides a UK-focused cost-effectiveness evaluation of implementing smoking cessation into cancer care in the NHS. The results show that over a lifetime time horizon, at a willingness-to-pay threshold of £20,000 per QALY gained, offering smoking cessation at cancer diagnosis is a cost-effective intervention in lung cancer, head and neck cancer, kidney cancer, and general cancer populations. In all four populations, offering smoking cessation at cancer diagnosis extends life by approximately 2–8 months and slows cancer progression by 2–6 months.

Overall, this analysis indicates that implementing smoking cessation services into NHS cancer care would provide large quality of life and survival benefits, and cost a small amount of money when compared with investments in expensive cancer treatment. Smoking cessation services align with the NHS 10-year plan to move from treating sickness to preventing illnesses and demonstrates a good value for money. The analysis highlights the importance of effective service integration to maximise health outcomes.

## Contributors

ME and JL led the initial conceptualisation for the study. MG, ETT, SH and RoM further conceptualised and developed the health economic analysis. ME, SA, MC, JL, RaM, GW and MS formed a clinical reference group to review the findings of the evaluation and make adaptations to the model as required. MG, ETT, SH and RoM developed the first manuscript draft and all authors provided expert review and amendments over several revisions to the final version.

## Data sharing statement

Data sharing is not applicable to this article as no datasets were generated or analysed during the current study.

## Declaration of interests

MG, ETT, SH and RoM are employees of York Health Economics Consortium; their institution received consulting fees from Manchester University NHS Foundation Trust for undertaking this research. In the past 36 months, GW received a grant in a related field by the National Cancer Institute, royalties from the Medical University of South Carolina in an unrelated field, consulting fees from the Canadian Partnership Against Cancer, payment/honoraria (speaker fees) from nonprofit organizations, participated in data safety monitoring/advisory boards for Varian and non-profit universities, and worked as a medical, research and expertise consultant. RaM’s university received grants from Yorkshire Cancer Research (NOT414; RA/2023/R2/113), Cancer Research UK (PICATR-2022/100010), the UK Prevention Research Partnership (MR/S037519/1) and Horizon 2020 (ID: 848294). RaM also received a travel bursary from the International Association for the Study of Lung Cancer for travelling to a conference, participated in a data safety monitory/advisory board for the University of Birmingham, is a Trustee for the Action on Smoking and Health, and is a member of the Royal College of Physicians’ Tobacco Advisory Group. In the past 36 months, MC has received a grant/contract from Yorkshire Cancer Research and the National Institute for Health Research.

Disclaimer: Where authors are identified as personnel of the International Agency for Research on Cancer/World Health Organization, the authors alone are responsible for the views expressed in this article and they do not necessarily represent the decisions, policy or views of the International Agency for Research on Cancer/World Health Organization.

## References

[1] Mullen K.A., Hurley K., Hewitson S. (2024). Cost-effectiveness of point of care smoking cessation interventions in oncology clinics. Br J Cancer.

[2] Zhu S., Gao J., Zhang L. (2025). Global, regional, and national cardiovascular disease burden attributable to smoking from 1990 to 2021: findings from the GBD 2021 study. Tob Induc Dis.

[3] Cancer Research UK Tobacco statistics 2025. https://www.cancerresearchuk.org/health-professional/cancer-statistics/risk/tobacco#heading-Zero.

[4] Eng L., Evans W.K., Eisele M. (2025). The International Association for the Study of Lung Cancer staging project: the impact of smoking status on lung cancer staging in the ninth edition of the TNM classification. J Thorac Oncol.

[5] Bergman M., Fountoukidis G., Smith D., Ahlgren J., Lambe M., Valachis A. (2022). Effect of smoking on treatment efficacy and toxicity in patients with cancer: a systematic review and meta-analysis. Cancers.

[6] Jassem J. (2019). Tobacco smoking after diagnosis of cancer: clinical aspects. Transl Lung Cancer Res.

[7] Chellappan S. (2022). Smoking cessation after cancer diagnosis and enhanced therapy response: mechanisms and significance. Curr Oncol.

[8] Nolazco J.I., Tang Y., Alkhatib K.Y., King A.J., Mossanen M., Chang S.L. (2023). Smoking status among cancer patients by medical specialty: a US nationwide representative analysis. Cancer Med.

[9] Sheikh M., Mukeriya A., Shangina O., Brennan P., Zaridze D. (2021). Postdiagnosis smoking cessation and reduced risk for lung cancer progression and mortality: a prospective cohort study. Ann Intern Med.

[10] Sheikh M., Mukeriya A., Zahed H. (2023). Smoking cessation after diagnosis of kidney cancer is associated with reduced risk of mortality and cancer progression: a prospective cohort study. J Clin Oncol.

[11] Cinciripini P.M., Kypriotakis G., Blalock J.A. (2024). Survival outcomes of an early intervention smoking cessation treatment after a cancer diagnosis. JAMA Oncol.

[12] Gemine R.E., Davies G.R., Lanyon K. (2023). Quitting smoking improves two-year survival after a diagnosis of non-small cell lung cancer. Lung Cancer.

[13] NHS England (2025). https://digital.nhs.uk/data-and-information/publications/statistical/statistics-on-nhs-stop-smoking-services-in-england.

[14] Williams P.J., Philip K.E., Alghamdi S.M. (2023). Strategies to deliver smoking cessation interventions during targeted lung health screening - a systematic review and meta-analysis. Chron Respir Dis.

[15] Evison M, Naylor R, Malcolm R (2026). Health economic model to evaluate the cost-effectiveness of smoking cessation services integrated within lung cancer screening in the United Kingdom. Thorax.

[16] National Institute for Health and Care Excellence (NICE) (2022). https://www.nice.org.uk/process/pmg36.

[17] Sheikh M., Nemati S., Dudding T. (2024). 882P quitting smoking after diagnosis of head and neck cancer is associated with reduced risk of mortality: a multicentric prospective cohort study. Ann Oncol.

[18] Hughes J.R., Peters E.N., Naud S. (2008). Relapse to smoking after 1 year of abstinence: a meta-analysis. Addict Behav.

[19] Gilpin E.A., Pierce J.P., Farkas A.J., Farkas A.J. (1997). Duration of smoking abstinence and success in quitting. J Natl Cancer Inst.

[20] Dobbie F., Hiscock R., Leonardi-Bee J. (2015). Evaluating long-term outcomes of NHS stop smoking services (ELONS): a prospective cohort study. Health Technol Assess.

[21] Therneau T.L.T., Atkinson E., Crowson C. Surival: survival analysis 2024. https://cran.r-project.org/web/packages/survival/index.html.

[22] (2023). WebPlotDigitizer.

[23] Guyot P.A.A., Ouwens M.J., Welton N.J. (2012). Enhanced secondary analysis of survival data: reconstructing the data from published Kaplan-Meier survival curves. BMC Med Res Methodol.

[24] Baio G.B.A., Cooney P., Jones A., Green N. (2023). https://CRAN.R-project.org/package=survHE.

[25] Jackson C.M.P., Amdahl J., Warkentin M.T., Sweeting M., Kunzmann K. (2023). https://cran.r-project.org/web/packages/flexsurv/index.html.

[26] National Institute for Health and Care Excellence (2010). https://www.ncbi.nlm.nih.gov/books/NBK310331/.

[27] Murray R.L., Alexandris P., Baldwin D. (2024). Uptake and 4-week quit rates from an opt-out co-located smoking cessation service delivered alongside community-based low-dose computed tomography screening within the Yorkshire Lung Screening Trial. Eur Respir J.

[28] Wu Q., Gilbert H., Nazareth I. (2018). Cost-effectiveness of personal tailored risk information and taster sessions to increase the uptake of the NHS stop smoking services: the Start2quit randomized controlled trial. Addiction.

[29] Livingstone-Banks J., Ordóñez-Mena J.M., Hartmann-Boyce J. (2019). Print-based self-help interventions for smoking cessation. Cochrane Database Syst Rev.

[30] National Institute for Health and Care Excellence (NICE) (2021).

[31] Lindson N., Theodoulou A., Ordóñez-Mena J.M. (2023). Pharmacological and electronic cigarette interventions for smoking cessation in adults: component network meta-analyses. Cochrane Database Syst Rev.

[32] Hawkins J., Hollingworth W., Campbell R. (2010). Long-term smoking relapse: a study using the british household panel survey. Nicotine Tob Res.

[33] Gummerson S.L.J., Taylor K., Lobo T., Jensen R. (2021). The characteristics of patients who quit smoking in the year following a cancer diagnosis. J Cancer Surviv.

[34] Royal College of Physicians (2018).

[35] Prescott E., Hippe M., Schnohr P., Hein H.O., Vestbo J. (1998). Smoking and risk of myocardial infarction in women and men: longitudinal population study. BMJ.

[36] Jones K., Weatherly H., Birch S. (2025).

[37] National Institute for Health and Care Excellence (NICE) (2025). https://bnf.nice.org.uk/.

[38] GOV.UK Drugs and pharmaceutical electronic market information tool (eMIT). https://www.gov.uk/government/publications/drugs-and-pharmaceutical-electronic-market-information-emit.

[39] NHS England National cost collection for the NHS. https://www.england.nhs.uk/costing-in-the-nhs/national-cost-collection/.

[40] Hernández Alava M.P., S Wailoo A. (2022).

[41] Office of National Statistics (2023). National and subnational mid-year population estimates for England and Wales by administrative area, age and sex.

[42] D’Angelo H., Hohl S.D., Rolland B. (2022). Reach and effectiveness of the NCI cancer moonshot-funded cancer center cessation initiative. Transl Behav Med.

[43] Landeiro F., Harris C., Groves D. (2024). The economic burden of cancer, coronary heart disease, dementia, and stroke in England in 2018, with projection to 2050: an evaluation of two cohort studies. Lancet Healthy Longev.

[44] Howard P., Knight C., Boler A., Baker C. (2008). Cost-utility analysis of varenicline versus existing smoking cessation strategies using the BENESCO Simulation model: application to a population of US adult smokers. Pharmacoeconomics.

[45] Lutz M.A., Lovato P., Cuesta G. (2012). Cost-effectiveness analysis of varenicline versus existing smoking cessation strategies in Central America and the Caribbean using the BENESCO model. Hosp Pract.

[46] Bolin K., Wilson K., Benhaddi H. (2009). Cost-effectiveness of varenicline compared with nicotine patches for smoking cessation—results from four European countries. Eur J Publ Health.

[47] National Institute for Health and Care Excellence (2022). https://www.nice.org.uk/guidance/ta798/documents/committee-papers-2.

[48] National Institute for Health and Care Excellence (2021). https://www.nice.org.uk/guidance/ta736/history.

[49] NHS, UK Government (2025).

[50] Buttery S.C., Williams P., Mweseli R. (2022). Immediate smoking cessation support versus usual care in smokers attending a targeted lung health check: the QuLIT trial. BMJ Open Respir Res.

[51] Williams P.J., Philip K.E.J., Buttery S.C. (2024). Immediate smoking cessation support during lung cancer screening: long-term outcomes from two randomised controlled trials. Thorax.

[52] Su Z., Xie Y., Huang Z. (2025). Second hand smoke attributable disease burden in 204 countries and territories, 1990-2021: a systematic analysis from the Global Burden of Disease Study 2021. Respir Res.

[53] Flor L.S., Anderson J.A., Ahmad N. (2024). Health effects associated with exposure to secondhand smoke: a Burden of proof study. Nat Med.

[54] Cancer Research UK (2025). https://www.cancerresearchuk.org/sites/default/files/cancer_in_the_uk_2025_socioeconomic_deprivation.pdf.

[55] Department of Health and Social Care (2025). https://fingertips.phe.org.uk/profile/tobacco-control/supporting-information/smokingandinequalities.

[56] Agrawal S., La Corte P.C., Frost T. (2025). Is the provision of a national opt-out tobacco dependence treatment service in acute hospitals in England equitable? A national cohort study. Thorax.

[57] Love-Koh J., Pennington B., Owen L., Taylor M., Griffin S. (2020). How health inequalities accumulate and combine to affect treatment value: a distributional cost-effectiveness analysis of smoking cessation interventions. Soc Sci Med.

[58] Action on Smoking and Health (ASH) (2023). https://ash.org.uk/uploads/Smoking-and-Surgery-Factsheet-2023.pdf?v=1682434565.

[59] Sheikh M. (2025).

[60] Audit N.L.C. (2024).

[61] Miquel Dominguez A., Tan E.H., Burn E. (2024). Incidence, prevalence, and survival of head and neck cancers in the United Kingdom from 2000-2021. medRxiv.

[62] Cancer Research UK Kidney cancer incidence statistics 2025. https://www.cancerresearchuk.org/health-professional/cancer-statistics/statistics-by-cancer-type/kidney-cancer/incidence#heading-Zero.

