# Supplementary material for: Economic and health impact of integrating smoking cessation services into NHS cancer care pathways in England: a modelling study
Source: Lancet Reg Health Eur. 2026 Sep 20;71:101869. doi: 10.1016/j.lanepe.2026.101869 (PMC13628226; doi:10.1016/j.lanepe.2026.101869)
Supplement: Supplementary file 1 [file mmc1.docx]

**Contents**

[Supplementary Material	4](#_Toc235787081)

[Time-to-event	4](#_Toc235787082)

[**Table S1:**	**Base case distributions for each population**	4](#_Toc235787083)

[Model parameters	4](#_Toc235787084)

[Population characteristics	4](#_Toc235787085)

[**Table S2:**	**Starting age inputs**	4](#_Toc235787086)

[**Table S3:**	**Proportion male inputs**	4](#_Toc235787087)

[Effectiveness	5](#_Toc235787088)

[**Table S4:**	**Proportion of people receiving different smoking cessation methods**	5](#_Toc235787089)

[**Table S5:**	**Probability of quitting smoking at 6 months**	5](#_Toc235787090)

[**Table S6:**	**Relative rate of quitting at 6 months versus behavioural support**	5](#_Toc235787091)

[**Table S7:**	**Relapse rate of those using smoking cessation**	5](#_Toc235787092)

[Comorbidities	6](#_Toc235787093)

[**Table S8:**	**Comorbidity relative risk**	7](#_Toc235787094)

[**Table S9:**	**Comorbidity costs and utility decrements**	7](#_Toc235787095)

[**Table S10:**	**Prevalence of smoking in males by age**	7](#_Toc235787096)

[**Table S11:**	**Prevalence of smoking in females by age**	8](#_Toc235787097)

[**Table S12:**	**Prevalence of COPD**	8](#_Toc235787098)

[**Table S13:**	**Prevalence of CHD**	8](#_Toc235787099)

[**Table S14:**	**Prevalence of MI**	8](#_Toc235787100)

[**Table S15:**	**Prevalence of type 2 diabetes**	9](#_Toc235787101)

[**Table S16:**	**Prevalence of stroke**	9](#_Toc235787102)

[**Table S17:**	**Prevalence of dementia**	9](#_Toc235787103)

[**Table S18:**	**Prevalence of asthma**	9](#_Toc235787104)

[Costs and resource use	10](#_Toc235787105)

[Smoking cessation costs	10](#_Toc235787106)

[**Table S19:**	**Unit cost of smoking cessation**	10](#_Toc235787107)

[**Table S20:**	**Purchase type**	10](#_Toc235787108)

[**Table S21:**	**NRT product weights**	10](#_Toc235787109)

[System Redesign Cost	11](#_Toc235787110)

[**Table S22:**	**System redesign cost**	11](#_Toc235787111)

[Lung Cancer Costs	11](#_Toc235787112)

[Progression treatment costs	11](#_Toc235787113)

[**Table S23:**	**Unit costs of lung cancer treatments**	11](#_Toc235787114)

[**Table S24:**	**Proportions having second line treatment**	12](#_Toc235787115)

[**Table S25:**	**Progression treatment costs and proportions**	12](#_Toc235787116)

[Health state costs	12](#_Toc235787117)

[**Table S26:**	**Health state resources (per month)**	12](#_Toc235787118)

[**Table S27:**	**Unit costs of health state resources**	13](#_Toc235787119)

[Head and Neck Cancer Costs	13](#_Toc235787120)

[Progression treatment costs	13](#_Toc235787121)

[**Table S28:**	**Unit costs of head and neck cancer treatments**	13](#_Toc235787122)

[**Table S29:**	**Proportions having second line treatment**	13](#_Toc235787123)

[**Table S30:**	**Progression treatment costs and proportions**	14](#_Toc235787124)

[Health state costs	14](#_Toc235787125)

[**Table S31:**	**Health state resources (per month)**	14](#_Toc235787126)

[**Table S32:**	**Unit costs of health state resources**	14](#_Toc235787127)

[Kidney Cancer Costs	14](#_Toc235787128)

[**Table S33:**	**Unit costs of Kidney cancer**	14](#_Toc235787129)

[**Table S34:**	**Proportions having second line treatment**	15](#_Toc235787130)

[**Table S35:**	**Progression treatment costs and proportions**	15](#_Toc235787131)

[Health state costs	15](#_Toc235787132)

[**Table S36:**	**Health state resources (per month)**	15](#_Toc235787133)

[**Table S37:**	**Unit costs of health state resources**	16](#_Toc235787134)

[General Cancer Costs	16](#_Toc235787135)

[**Table S38:**	**The total costs for general cancer**	16](#_Toc235787136)

[Administration cost	16](#_Toc235787137)

[End of life cost	16](#_Toc235787138)

[Health-related quality of life	16](#_Toc235787139)

[**Table S39:**	**Cancer-related utility values**	16](#_Toc235787140)

[**Table S40:**	**Demographic information to information adjustment factors**	17](#_Toc235787141)

[**Table S41:**	**Smoking-related disutility values**	17](#_Toc235787142)

[Results: Deterministic base case results	17](#_Toc235787143)

[**Table S42:**	**Per-person cost breakdown for the lung cancer population**	17](#_Toc235787144)

[**Table S43:**	**Per-person cost breakdown for the head and neck cancer population**	18](#_Toc235787145)

[**Table S44:**	**Per-person cost breakdown for the kidney cancer population**	18](#_Toc235787146)

[**Table S45:**	**Per-person cost breakdown for the general cancer population**	19](#_Toc235787147)

[**Table S46:**	**Costs per-person as a proportion of the total for each model arm**	19](#_Toc235787148)

[Results: Deterministic sensitivity analysis (DSA)	20](#_Toc235787149)

[**Figure S1:**	**Tornado diagram for Lung cancer**	21](#_Toc235787150)

[**Figure S2:**	**Tornado diagram for Head and neck cancer**	21](#_Toc235787151)

[**Figure S3:**	**Tornado diagram for Kidney cancer**	22](#_Toc235787152)

[**Figure S4:**	**Tornado diagram for General cancer**	22](#_Toc235787153)

[Results: Probabilistic sensitivity analysis (PSA)	23](#_Toc235787154)

[Lung cancer	23](#_Toc235787155)

[**Table S47:**	**PSA mean outcomes per person for the lung cancer population**	23](#_Toc235787156)

[Head and neck cancer	23](#_Toc235787157)

[**Table S48:**	**PSA mean outcomes per person for the head and neck cancer population**	23](#_Toc235787158)

[Kidney cancer	24](#_Toc235787159)

[**Table S49:**	**PSA mean outcomes per person for the kidney cancer population**	24](#_Toc235787160)

[General cancer	24](#_Toc235787161)

[**Table S50:**	**PSA mean outcomes per person for the general cancer population**	24](#_Toc235787162)

[Results: threshold analysis	25](#_Toc235787163)

[**Figure S5:**	**Threshold analysis of smoking cessation uptake for lung cancer population**	25](#_Toc235787164)

[**Figure S6:**	**Threshold analysis of smoking cessation uptake for head and neck cancer population**	25](#_Toc235787165)

[**Figure S7:**	**Threshold analysis of smoking cessation uptake for kidney cancer population**	26](#_Toc235787166)

[**Figure S8:**	**Threshold analysis of smoking cessation uptake for general cancer population**	26](#_Toc235787167)

[**Figure S9:**	**Threshold analysis of e-consultation cost for lung cancer population**	27](#_Toc235787168)

[**Figure S10:**	**Threshold analysis of e-consultation cost for head and neck cancer population**	27](#_Toc235787169)

[**Figure S11:**	**Threshold analysis of e-consultation cost for kidney cancer population**	28](#_Toc235787170)

[**Figure S12:**	**Threshold analysis of e-consultation cost for general cancer population**	28](#_Toc235787171)

[Results: scenario analysis	29](#_Toc235787172)

[Scenario 1: Using different parametric models	29](#_Toc235787173)

[Scenario 2: Using the kidney cancer KM data to calculate the hazard ratios for PFS	29](#_Toc235787174)

[**Table S51:**	**Hazard ratios for quit and continued smoking for OS versus PFS**	29](#_Toc235787175)

[**Table S52:**	**Per-person summary results for scenario 2 as compared to the base case**	29](#_Toc235787176)

[Scenario 3: Time horizon	30](#_Toc235787177)

[**Table S53:**	**Results across different time horizons**	30](#_Toc235787178)

[Scenario 4: Benefits of smoking cessation for cancer outcomes	30](#_Toc235787179)

[**Table S54:**	**Per-person summary results for scenario 4 as compared to the base case**	31](#_Toc235787180)

[Scenario 5: Benefits of smoking cessation for smoking-related illnesses	31](#_Toc235787181)

[**Table S55:**	**Per-person summary results for scenario 5 as compared to the base case**	31](#_Toc235787182)

[Scenario 6: Equal treatments given across pathways	31](#_Toc235787183)

[**Table S56:**	**Per-person summary results for scenario 6 as compared to the base case**	32](#_Toc235787184)

[Scenario 7: Smoking cessation intervention	32](#_Toc235787185)

[**Table S57:**	**Per-person summary results for scenario 7 as compared to used 100% behavioural support**	33](#_Toc235787186)

[Scenario 8: Smoking cessation intervention	33](#_Toc235787187)

[**Table S58:**	**Per-person summary results for scenario 8 as compared to the base-case lung cancer population results**	33](#_Toc235787188)

[References	34](#_Toc235787189)

# Supplementary Material

Time-to-event

From the modelled long-term extrapolations, coefficients were produced and included in the model to calculate long-term overall survival (OS) and progression-free survival (PFS) for both quit and continued smoking populations in each of the four subpopulations. The chosen distributions for each of the datasets is provided in Table S1.

### **Table S1: Base case distributions for each population**

| **Population** | **Curve** | **Smoking status** | Distribution selected in base case |
| --- | --- | --- | --- |
| Lung cancer | Overall survival | Quit smoking | Log-normal |
|  |  | Continued smoking | Log-normal |
|  | Progression-free survival | Quit smoking | Weibull |
|  |  | Continued smoking | Weibull |
| Head and neck cancer | Overall survival | Quit smoking | Generalised gamma |
|  |  | Continued smoking | Generalised gamma |
|  | Progression-free survival | Quit smoking | Hazard ratio of OS versus PFS |
|  |  | Continued smoking | Hazard ratio of OS versus PFS |
| Kidney cancer | Overall survival | Quit smoking | Exponential |
|  |  | Continued smoking | Exponential |
|  | Progression-free survival | Quit smoking | Exponential |
|  |  | Continued smoking | Exponential |
| General cancer | Overall survival | Quit smoking | Generalised gamma |
|  |  | Continued smoking | Generalised gamma |
|  | Progression-free survival | Quit smoking | Hazard ratio of OS versus PFS |
|  |  | Continued smoking | Hazard ratio of OS versus PFS |

Abbreviations: OS – overall survival; PFS – progression-free survival.

Model parameters

## Population characteristics

Table S2 and Table S3 outline the starting ages and proportion male inputs used in the model for the four populations. The age and percentage male parameters impacted key aspects of the cost-effectiveness model, such as background mortality, quality of life parameters (via adjustment factors), and the time horizon length.

### **Table S2: Starting age inputs**

| Parameters | Average age from KM data | Starting age (used in model) | Source for starting age  (from the literature) |
| --- | --- | --- | --- |
| Lung cancer | 61 years old | 74·0 years old | National Lung Cancer Audit 2024 ^1^ |
| Head and neck cancer | 59 years old | 64·0 years old | Dominguez et al., 2025 ^2^ |
| Kidney cancer | 56 years old | 64·0 years old | Kidney Cancer Research UK 2025 ^3^ |
| General cancer | 55 years old | 69·0 years old | Average of lung and head and neck. |

Abbreviations: KM – Kaplan-Meier.

### **Table S3: Proportion male inputs**

| Parameters | Proportion male from KM data | Proportion male (used in model) | Source (from the literature) |
| --- | --- | --- | --- |
| Lung cancer | 88·5% | 50·2% | National Lung Cancer Audit 2024 ^1^ |
| Head and neck cancer | 83·0% | 69·2% | Dominguez et al., 2025 ^2^ |
| Kidney cancer | 80·0% | 63·5% | Cancer Research UK 2025 ^4^ |
| General cancer | 52·2% | 59·7% | Average of lung and head and neck. |

Abbreviations: KM – Kaplan-Meier.

## Effectiveness

The proportion of people receiving different smoking cessation methods when offered at diagnosis and for standard of care is presented in Table S4. The base case accounts for differences in smoking cessation treatment offered, given that previous evidence suggests that decentralised services are more likely to use pharmacological interventions.

### **Table S4: Proportion of people receiving different smoking cessation methods**

| **Parameters** | Proportion | Source |
| --- | --- | --- |
| **Treatments offered at diagnosis programme** | | |
| Behavioural support alone | 14·58% | Murray et al., 2024 ^5^. Although this was specific to a study setting, anecdotally, clinicians have provided feedback E-Cigarettes are still being used as a cessation tool. |
| Cytisine | 3·65% |  |
| Varenicline | 3·65% |  |
| NRT I/s | 12·50% |  |
| NRT I&s | 12·50% |  |
| Nicotine containing E-cigarettes | 18·75% |  |
| Varenicline + NRT I/s | 0·00% |  |
| E-cigarettes + NRT I/s | 34·38% |  |
| **Treatments offered in usual care programme** | | |
| Behavioural support alone | 57·50% | Williams et al., 2023 ^6^ |
| Cytisine | 0·00% |  |
| Varenicline | 2·70% |  |
| NRT I/s | 5·50% |  |
| NRT I&s | 3·80% |  |
| Nicotine containing E-cigarettes | 30·50% |  |
| Varenicline + NRT I/s | 0·00% |  |
| E-cigarettes + NRT I/s | 0·00% |  |

Abbreviations: NRT – nicotine replacement therapy.

The probability of quitting smoking at six months, including the natural quit rate and the proportion quitting with behavioural support is detailed in Table S5. The relative rate of quitting at 6 months for alternative smoking cessation interventions compared with behavioural support are detailed in Table S6. The relapse rate when using smoking cessation between 6 months to 5 years is detailed Table S7.

### **Table S5: Probability of quitting smoking at 6 months**

| Parameter | Probability | Source |
| --- | --- | --- |
| Natural quit probability | 5·00% | Livingstone-Banks et al., 2019 ^7^ |
| Proportion quitting with behavioural support | 11·49% | NG209, NICE guideline 2021 ^8^ |

### **Table S6: Relative rate of quitting at 6 months versus behavioural support**

| Parameter | Relative risk | Source |
| --- | --- | --- |
| Cytisine | 2·06 | Lindson et al., 2023 ^9^ |
| Varenicline | 2·27 | NG209, NICE guideline 2021 ^8^ |
| NRT I/s | 1·83 |  |
| NRT I&s | 2·71 |  |
| E-cigarettes | 2·25 |  |
| Varenicline & NRT I/s | 2·58 |  |
| E-cigarettes & NRT I/s | 2·93 |  |

Abbreviations: NRT – nicotine replacement therapy.

### **Table S7: Relapse rate of those using smoking cessation**

| **Parameter** | Relapse proportion | Source |
| --- | --- | --- |
| Between 6 months and 1 year | 14·40% | NG209, NICE guideline 2021^8^ |
| Between 1 year and 2 years | 7·90% | Hawkins et al., 2010 ^10^ |
| Between 2 years and 3 years | 4·90% |  |
| Between 3 years and 4 years | 3·00% |  |
| Between 4 years and 5 years | 2·30% |  |

Abbreviations: NRT – nicotine replacement therapy.

## Comorbidities

The relative risk of comorbidities for current smokers and former smokers are presented in Table S8. The prevalence of smoking by gender and age is displayed in Table S9 and Table S10. Each table is stratified by age categories, ranging from 12 to 15 years up to 75+ years, and by smoking status.

The prevalence of chronic obstructive pulmonary diseases (COPD), coronary heart disease (CHD), myocardial infarction (MI), type 2 diabetes and stroke, stratified by gender and age is shown in Table S10 to Table S16. No data was reported for the 12-to-15-year age category; thus, prevalence was assumed to be 0%. Additionally, gender specific data was unavailable for COPD; therefore, prevalence was assumed to be equal for both genders.

The prevalence of dementia by gender and age is presented in Table S17. As no data was reported for ages 12 to 54 years, prevalence was assumed to be 0%. The reported prevalence data began at age 60; therefore, it was assumed that the prevalence would extend to age category 55 to 59 years.

Finally, the prevalence of asthma by gender and age is detailed in Table S18. The reported data age started at 18 to 34 years old; therefore, it was assumed that the prevalence would be the same for the 12-to-15-year age category.

The method for estimating the non-smoker prevalence is provided below:

The prevalence of each comorbidity was adjusted to remove the impact of smoking (current and former). This impact was estimated as the prevalence of smoking (current and former) multiplied by the relative risk of the relevant comorbidity. The following calculation was used to estimate the adjusted comorbidity prevalence per age and gender:

$$NScp=\frac{GPcp}{(NSp+\left( FSp x FSrr \right)+(CSp x CSrr)}$$

Where:

- NS is ‘non-smoker’
- FS is ‘former smoker’
- CS is ‘current smoker’
- GP is ‘general population’
- cp is ‘comorbidity prevalence’
- p is ‘prevalence’
- rr is ‘comorbidity relative risk’

### **Table S8: Comorbidity relative risk**

| Parameter | Relative risk for current smoker | Relative risk for former smoker | Source |
| --- | --- | --- | --- |
| COPD | 4·01 | 3·13 | Royal College of Physicians 2018 ^11^ |
| CHD: Males | 1·96 | 1·16 |  |
| CHD: Females | 1·95 | 1·37 |  |
| MI: Males | 1·43 | 1·11 | Prescott et al., 1998 ^12^ |
| MI: Females | 2·24 | 1·05 |  |
| Type 2 Diabetes | 1·37 | 1·14 | Royal College of Physicians 2018 ^11^ |
| Stroke: Males | 1·57 | 1·08 |  |
| Stroke: Females | 1·83 | 1·17 |  |
| Dementia | 1·30 | 1·01 |  |

Abbreviations: CHD – coronary heart disease; COPD – chronic obstructive pulmonary disease; MI – myocardial infarction.

### **Table S9: Comorbidity costs and utility decrements**

| Parameter | Annual costs | Disutility | Source |
| --- | --- | --- | --- |
| COPD | £2,003 | 0·12 | Cost: Punekar et al., 2014 ^13^  Disutility: Hernandez Alava et al., 2022 ^14^, Nolan et al., 2016 ^15^ |
| CHD | £6,648 | 0·14 | Cost: Landerio et al., 2024 ^16^  Disutility: Hernandez Alava et al., 2022 ^14^, Goldsmith et al., 2010 ^17^ |
| MI | £3,133 | 0·04 | Cost: Danese et al., 2016 ^18^  Disutility: Hernandez Alava et al., 2022 ^14^, Munyombwe et al., 2021 ^19^ |
| Type 2 Diabetes | £2,421 | 0·02 | Cost: Average between NHS England 2019 ^20^ and Stedman et al., 2020 ^21^  Disutility: Boye et al., 2022 ^22^ |
| Stroke | £7,451 | 0·15 | Cost: Xu et al., 2018 ^23^ Disutility: Luengo-Fernandez et al., 2013 ^24^ |
| Dementia | £6,833 | 0·14 | Cost: Alzheimer’s Society 2014 ^25^. Disutility: Romback et al., 2021 ^26^ |
| Asthma | £1,560 | 0·02 | Cost: Leaviss et al., 2014 ^27^. Disutility: Hernandez Alava et al., 2022 ^14^, Sullivan et al., 2016 ^28^ |

Abbreviations: CHD – coronary heart disease; COPD – chronic obstructive pulmonary disease; MI – myocardial infarction; NHS – National Health Service.

### **Table S10: Prevalence of smoking in males by age**

| **Age** | Non-smoker | Former smoker | Current smoker | Source |
| --- | --- | --- | --- | --- |
| 12 to 15 | 94·84% | 2·16% | 3·00% | Current smoker source: NHS England 2024 ^29^  Non-smoking and former smoker source: Action of Smoking and Health 2025 ^30^ |
| 16 to 24 | 80·71% | 8·06% | 11·22% | NHS England 2024 ^29^ |
| 25 to 34 | 61·34% | 17·89% | 20·76% |  |
| 35 to 44 | 59·42% | 23·79% | 16·78% |  |
| 45 to 54 | 51·80% | 29·48% | 18·72% |  |
| 55 to 64 | 57·65% | 29·97% | 12·39% |  |
| 65 to 74 | 55·26% | 34·64% | 10·10% |  |
| 75+ | 52·63% | 42·58% | 4·80% |  |

Abbreviations: NHS – National Health Service.

### **Table S11: Prevalence of smoking in females by age**

| **Age** | Non-smoker | Former smoker | Current smoker | Source |
| --- | --- | --- | --- | --- |
| 12 to 15 | 94·05% | 1·95% | 4.00% | Current smoker source: NHS England 2024 ^29^  Non-smoking and former smoker source: Action of Smoking and Health 2025 ^30^ |
| 16 to 24 | 88·54% | 3·75% | 7·70% | NHS England 2024 ^29^ |
| 25 to 34 | 72·63% | 12·46% | 14·91% |  |
| 35 to 44 | 70·42% | 18·64% | 10·93% |  |
| 45 to 54 | 62·66% | 24·01% | 13·33% |  |
| 55 to 64 | 64·02% | 24·22% | 11·75% |  |
| 65 to 74 | 58·93% | 30·93% | 10·15% |  |
| 75+ | 62·91% | 31·90% | 5·19% |  |

Abbreviations: NHS – National Health Service.

### **Table S12: Prevalence of COPD**

| **Age** | Male | Female | Source |
| --- | --- | --- | --- |
| 12 to 15 | 0·00% | 0·00% | Stone et al., 2023 ^31^ |
| 16 to 24 | 1·28% | 1·28% |  |
| 25 to 34 | 1·28% | 1·28% |  |
| 35 to 44 | 1·96% | 1·96% |  |
| 45 to 54 | 4·15% | 4·15% |  |
| 55 to 64 | 4·15% | 4·15% |  |
| 65 to 74 | 8·31% | 8·31% |  |
| 75+ | 8·94% | 8·94% |  |

### **Table S13: Prevalence of CHD**

| Age | Male | Female | Source |
| --- | --- | --- | --- |
| 12 to 15 | 0·00% | 0·00% | British Heart Foundation 2024 ^32^ |
| 16 to 24 | 0·02% | 0·01% |  |
| 25 to 34 | 0·06% | 0·03% |  |
| 35 to 44 | 0·36% | 0·14% |  |
| 45 to 54 | 1·95% | 0·73% |  |
| 55 to 64 | 6·06% | 2·32% |  |
| 65 to 74 | 12·88% | 5·40% |  |
| 75+ | 25·11% | 14·68% |  |

### **Table S14: Prevalence of MI**

| Age | Male | Female | Source |
| --- | --- | --- | --- |
| 12 to 15 | 0·00% | 0·00% | NHS England 2018 ^33^ |
| 16 to 24 | 0·00% | 0·08% |  |
| 25 to 34 | 0·00% | 0·08% |  |
| 35 to 44 | 0·34% | 0·00% |  |
| 45 to 54 | 3·15% | 1·03% |  |
| 55 to 64 | 4·49% | 0·93% |  |
| 65 to 74 | 11·64% | 3·40% |  |
| 75+ | 12·90% | 5·08% |  |

Abbreviations: NHS – National Health Service.

### **Table S15: Prevalence of type 2 diabetes**

| Age | Male | Female | Source |
| --- | --- | --- | --- |
| 12 to 15 | 0·00% | 0·00% | NHS England 2020 ^34^ |
| 16 to 24 | 1·00% | 0·00% |  |
| 25 to 34 | 1·00% | 1·00% |  |
| 35 to 44 | 3·00% | 3·00% |  |
| 45 to 54 | 9·00% | 4·00% |  |
| 55 to 64 | 13·00% | 9·00% |  |
| 65 to 74 | 21·00% | 12·00% |  |
| 75+ | 18·00% | 15·00% |  |

Abbreviations: NHS – National Health Service.

### **Table S16: Prevalence of stroke**

| Age | Male | Female | Source |
| --- | --- | --- | --- |
| 12 to 15 | 0·11% | 0·11% | Bhatnagar et al., 2015 ^35^ |
| 16 to 24 | 0·11% | 0·11% |  |
| 25 to 34 | 0·11% | 0·11% |  |
| 35 to 44 | 0·11% | 0·11% |  |
| 45 to 54 | 0·89% | 0·79% |  |
| 55 to 64 | 2·69% | 1·96% |  |
| 65 to 74 | 6·40% | 4·39% |  |
| 75+ | 14·89% | 12·43% |  |

### **Table S17: Prevalence of dementia**

| Age | Male | Female | Source |
| --- | --- | --- | --- |
| 12 to 15 | 0·.00% | 0·00% | Alzheimer’s Society 2014 ^25^ |
| 16 to 24 | 0·00% | 0·00% |  |
| 25 to 34 | 0·00% | 0·00% |  |
| 35 to 44 | 0·00% | 0·00% |  |
| 45 to 54 | 0·00% | 0·00% |  |
| 55 to 64 | 0·90% | 0·90% |  |
| 65 to 69 | 1·50% | 1·80% |  |
| 70 to 74 | 3·10% | 3·00% |  |
| 75 to 79 | 5·30% | 6·60% |  |
| 80 to 84 | 10·30% | 11·70% |  |
| 85 to 89 | 15·10% | 20·20% |  |
| 90 to 94 | 22·60% | 33·00% |  |
| 95+ | 28·80% | 44·20% |  |

### **Table S18: Prevalence of asthma**

| Age | Current smoker | | Former smoker | | Source |
| --- | --- | --- | --- | --- | --- |
|  | Male | Female | Male | Female |  |
| 12 to 15 | 0·08% | 0·08% | 0·05% | 0·06% | Leaviss et al., 2014 ^27^ |
| 16 to 24 | 0·08% | 0·08% | 0·05% | 0·06% |  |
| 25 to 34 | 0·08% | 0·08% | 0·05% | 0·06% |  |
| 35 to 44 | 0·05% | 0·05% | 0·05% | 0·05% |  |
| 45 to 54 | 0·05% | 0·05% | 0·05% | 0·05% |  |
| 55 to 64 | 0·05% | 0·05% | 0·05% | 0·05% |  |
| 65 to 74 | 0·07% | 0·06% | 0·06% | 0·06% |  |
| 75+ | 0·07% | 0·06% | 0·06% | 0·06% |  |

Costs and resource use

## Smoking cessation costs

The unit costs of smoking cessation interventions are presented in Table S19. Any dated costs were inflated using the Personal Social Services Research Unit 2024 inflation indices.^36^ Nicotine replacement therapy (NRT) and e-cigarettes are available for purchase via prescription or over the counter. The proportions of purchase location for these two smoking cessation methods are presented in Table S20. National Health Service (NHS) England 2025 state that e-cigarettes are not currently available from the NHS on prescription; ^37^ therefore, 0% of e-cigarettes are obtained from prescription. The over-the-counter proportion was calculated as the remainder for both NRT and e-cigarettes. In the base case analysis, only the costs where NRTs or e-cigarettes had been purchased via prescription (healthcare costs) were included. The weighting of NRT products, stratified by short-acting and long-acting, is presented Table S21.

### **Table S19: Unit cost of smoking cessation**

|  | **Unit costs** | **Source** |
| --- | --- | --- |
| Behavioural support | £114·81 | Li et al., 2020 ^38^ |
| Cytisine | £115·00 | British National Formulary 2025 ^39^ |
| Varenicline | £139·21 | British National Formulary 2025 ^39^ |
| **NRT product costs** | | |
| NRT patch | £114·45 | British National Formulary 2025 ^39^, NHS Business Services Authority 2024 ^40^ |
| NRT lozenge | £55·86 | Schnoll et al., 2011 ^41^, NHS Business Services Authority 2024 ^40^, British National Formulary 2025 ^39^ |
| NRT gum | £98·52 | NHS Business Services Authority 2024 ^40^, British National Formulary 2025 ^39^ |
| NRT spray | £138·29 | Nicorette 2013 ^42^, NHS Business Services Authority 2024 ^40^, Office of National Statistics 2024 ^43^, NHS South London and Maudsley 2020 ^44^ |
| NRT inhalator | £374·22 | Nicorette 2023 ^45^, British National Formulary 2025 ^39^ |
| Any short acting NRT | £164·94 | NHS Business Services Authority 2024 ^40^ |
| **E-cigarette unit costs** | | |
| Starter pack cost | £33·98 | UK ECIG Store ^46^ |
| E-liquid cost | £223·44 | UK ECIG Store ^47^ |

Abbreviations: NHS – National Health Service; NRT – nicotine replacement therapy; UK – United Kingdom.

### **Table S20: Purchase type**

| Purchase location | Via prescription | Source |
| --- | --- | --- |
| NRT | 42·45% | Hajek et al., 2019 ^48^ |
| E-cigarettes | 0·00% | NHS England 2025 ^37^ |

Abbreviations: NHS – National Health Service; NRT – nicotine replacement therapy.

### **Table S21: NRT product weights**

| NRT product | NRT I/s | NRT I&s | Source |
| --- | --- | --- | --- |
| NRT patch | 17·99% | - | NRT I/s – NHS Business Services Authority 2024 ^40^  NRT I&s – NICE NG209 ^8^ |
| NRT lozenge | 2·54% | 0·00% |  |
| NRT gum | 11·68% | 25·45% |  |
| NRT spray | 64·74% | 50·72% |  |
| NRT inhalator | 3·06% | 5·74% |  |
| Any short acting NRT | - | 18·09% |  |

Abbreviations: NHS – National Health Service; NICE – National Institute for Health and Care Excellence; NRT – nicotine replacement therapy.

## System Redesign Cost

It was assumed that there would be a digital cost associated with restructuring smoking cessation in the NHS. However, this cost is currently unknown and is difficult to determine. Therefore, the cost of an e-consultation was used as a proxy, to account for the fact that it would cost to restructure NHS systems to implement smoking cessation into cancer care.

The system redesign cost for an e-consultation is presented in Table S22. It was assumed that there would be one e-consultation per person.

### **Table S22: System redesign cost**

| e-consultation | Value | Source |
| --- | --- | --- |
| Number of e-consultations per person | 1 | Assumption |
| Cost per e-consultation | £45·98 | Jones et al., 2024 ^36^ |

Lung Cancer Costs

## Progression treatment costs

Unit costs of lung cancer treatment are detailed in Table S23. These costs were calculated using pack and treatment unit inputs, including the pack cost, unit size, pack size, dose, cycle length and duration.

The proportions who receive second-line treatment are reported in Table S24. These proportions were split by those who received surgery (resectable) and those who do not receive surgery (non-resectable) at first line. The resectable proportions were also split by those who had locoregional recurrence (LRR) and disease metastasis (DM). Locoregional recurrence refers to the cancer being local to where it originally started (i.e. the lungs), and disease metastasis refers to people whose cancer has spread away from where it started.

The unit cost of treatment and the proportions receiving treatment were multiplied together to determine the total average cost of resectable and non-resectable treatment (reported in Table S25). The resectable and non resectable costs were then multiplied by the proportions that have surgery at first line to calculate the total one-off cost of progression of £28,491.

### **Table S23: Unit costs of lung cancer treatments**

| **Parameter** | Unit cost | Source |
| --- | --- | --- |
| Nivolumab | £141,335 | Calculated using data from the British National Formulary 2025 ^39^ and NICE TA798 ^49^ |
| Pembrolizumab | £38,506 |  |
| Durvalumab | £31,693 |  |
| Cisplatin | £1,192 | Calculated using data from the eMIT ^50^ and NICE TA798 ^49^ |
| Carboplatin | £1,141 |  |
| Docetaxel | £1,024 |  |
| Erlotinib | £1,109 |  |
| Pemetrexed | £1,949 |  |
| Gemcitabine | £2,302 |  |
| Paclitaxel | £1,173 |  |
| Afatinib | £24,194 | Calculated using data from the British National Formulary 2025 ^39^ and NICE TA798 ^49^ |
| PDC makeup | £3,141 | Based on NICE TA1043 ^51^ and TA798 ^49^, it was assumed that PDC was comprised of cisplatin and pemetrexed. |
| Pembrolizumab + paclitaxel + carboplatin | £40,820 | Calculated – sum of the Pembrolizumab, paclitaxel and carboplatin costs |
| Salvage surgery | £11,329 | National Cost Collection. 2023/24 ^52^  Weighted average of DZ02H, DZ02J and DZ02K |
| Radiotherapy | £5,793 | National Cost Collection. 2023/24 ^52^  Weighted average of radical radiotherapy costs and CHARD costs |
| No treatment | £0 | Assumption |

Abbreviations: eMIT – the drugs and pharmaceutical electronic market information tool; NICE – National Institute for Health and Care Excellence; PDC – platinum doublet chemotherapy.

### **Table S24: Proportions having second line treatment**

| **Parameters** | Non resectable | Resectable | |
| --- | --- | --- | --- |
|  |  | Locoregional recurrence | Disease Metastasis |
| Nivolumab | 21·68% | 0·00% | 0·00% |
| Pembrolizumab | 5·59% | 0·00% | 25·00% |
| Durvalumab | 4·20% | 0·00% | 0·00% |
| Cisplatin | 9·79% | 0·00% | 0·00% |
| Carboplatin | 32·17% | 0·00% | 0·00% |
| Docetaxel | 16·08% | 0·00% | 0·00% |
| Erlotinib | 7·69% | 0·00% | 0·00% |
| Pemetrexed | 17·48% | 0·00% | 0·00% |
| Gemcitabine | 19·58% | 0·00% | 0·00% |
| Paclitaxel | 11·19% | 0·00% | 0·00% |
| Afatinib | 5·59% | 0·00% | 0·00% |
| PDC makeup | 0·00% | 58·00% | 25·00% |
| Pembrolizumab + paclitaxel + carboplatin | 0·00% | 0·00% | 25·00% |
| Salvage surgery | 0·00% | 17·50% | 0·00% |
| Radiotherapy | 35·66% | 30·00% | 0·00% |
| No treatment | 0·00% | 22·50% | 25·00% |

Abbreviations: PDC – platinum doublet chemotherapy.

### **Table S25: Progression treatment costs and proportions**

| **Parameter** | Value | Source |
| --- | --- | --- |
| Proportion that have surgery at first line (resectable) | 41·00% | NHS England ^53^ |
| Proportion that do not have surgery at first line (non-resectable) | 59·00% |  |
| Progressions that are locoregional recurrence | 50·00% | Assumption |
| Cost of treatment for resectable | £13,079 | Calculated |
| Cost of treatment for non-resectable | £39,200 |  |
| **Total one-off cost of progression** | **£28,491** |  |

Abbreviations: NHS – National Health Service.

## Health state costs

The monthly progression-free and progressed health state resources (Table S26) were applied to the unit costs (Table S27) associated with each of these resources. These costs were then summed to calculate the total monthly progression-free cost and total monthly progressed cost, which were £301.28 and £981.45, respectively. These monthly costs were then applied to those in the progression-free or progressed health states each cycle within the model.

### **Table S26: Health state resources (per month)**

| **Parameter** | Progression-free resource use | Progression resource use | Source |
| --- | --- | --- | --- |
| Specialist nurse appointment | 0·00 | 1·00 | NICE TA876 ^54^ |
| Oncologist appointment | 0·19 | 0·69 |  |
| Surgeon appointment | 0·14 | 0·20 |  |
| Other specialist visit | 0·33 | 0·33 |  |
| Hospitalisation | 0·02 | 0·08 |  |
| Emergency department visit | 0·06 | 0·13 |  |
| CT scan | 0·17 | 0·25 |  |
| Magnetic resonance imaging | 0·00 | 0·08 |  |
| Positron emission tomography scan | 0·02 | 0·17 |  |
| Electrocardiogram | 0·08 | 0·08 |  |
| Complete blood count | 1·44 | 5·77 |  |
| Metabolic panel, liver and renal function tests | 1·44 | 5·77 |  |

Abbreviations: CT – computerised tomography; NICE – National Institute for Health and Care Excellence.

### **Table S27: Unit costs of health state resources**

| **Parameter** | Unit cost | Source |
| --- | --- | --- |
| Specialist nurse appointment | £157·66 | National Cost Collection 2023/24 ^52^ |
| Oncologist appointment | £193·39 |  |
| Surgeon appointment | £218·31 |  |
| Other specialist visit | £193·39 |  |
| Hospitalisation | £2,465·50 |  |
| Emergency department visit | £323·48 |  |
| CT scan | £233·39 |  |
| Magnetic resonance imaging | £136·33 |  |
| Positron emission tomography scan | £614·82 |  |
| Electrocardiogram | £117·99 |  |
| Complete blood count | £6·95 |  |
| Metabolic panel, liver and renal function tests | £20·85 | Assumed to be equal to the cost of a cost of a complete blood count.  National Cost Collection 2023/24 ^52^ |

Abbreviations: CT – computerised tomography.

Head and Neck Cancer Costs

## Progression treatment costs

Unit costs of head and neck cancer treatment are detailed in Table S28. These costs have been calculated using pack and treatment unit inputs, including the pack cost, unit size, pack size, dose, cycle length and duration.

The proportions who receive second line treatment are reported in Table S29. These proportions were split by those who receive pembrolizumab and those who receive chemotherapy at first-line treatment. The unit cost of treatment and the proportions receiving treatment were multiplied together to determine the total cost of pembrolizumab and chemotherapy (reported in Table S30). The costs of pembrolizumab and chemotherapy were then applied to the proportion treated with pembrolizumab at first line to determine the one-off cost of treatment for progression of head and neck cancer (see Table S30).

### **Table S28: Unit costs of head and neck cancer treatments**

| **Parameter** | Unit cost | Source |
| --- | --- | --- |
| Docetaxel | £2,568 | Calculated using data from the eMIT ^50^ and NICE TA661 ^55^ |
| Nivolumab | £6,873 | Calculated using data from the British National Formulary 2025 ^39^ and NICE TA661 ^55^ |
| Paclitaxel | £1,268 | Calculated using data from the eMIT ^50^ and NICE TA661 ^55^ |
| Methotrexate | £5,236 |  |
| Carboplatin and paclitaxel | £1,923 |  |

Abbreviations: eMIT – the drugs and pharmaceutical electronic market information tool; NICE – National Institute for Health and Care Excellence.

### **Table S29: Proportions having second line treatment**

| Parameter | Proportions | | Source |
| --- | --- | --- | --- |
|  | Pembrolizumab at first line | Chemotherapy at first line |  |
| Docetaxel | 12·50% | 20·20% | NICE TA661 ^55^ |
| Nivolumab | 3·57% | 22·52% |  |
| Paclitaxel | 10·71% | 21·19% |  |
| Methotrexate | 7·50% | 0·33% |  |
| Carboplatin and paclitaxel | 17·14% | 9·27% |  |

Abbreviations: NICE – National Institute for Health and Care Excellence.

### **Table S30: Progression treatment costs and proportions**

| **Parameter** | Value | Source |
| --- | --- | --- |
| Proportion treated with pembrolizumab at first line | 50·00% | Assumption |
| Cost of pembrolizumab | £1,425 | Calculated |
| Cost of Chemotherapy | £2,531 |  |
| **Total one-off cost of progression** | **£1,978** |  |

## Health state costs

The monthly progression-free and progressed health state resources (Table S31) were applied to the unit costs associated with each of these resources (Table S32). These costs were then summed to calculate the total monthly progression-free cost and total monthly progression cost, which were £349·63 and £69·68, respectively. These monthly costs were then applied to those in the progression-free or progressed health states each cycle within the model.

### **Table S31: Health state resources (per month)**

| Parameter | Health state resources (per month) | | Source |
| --- | --- | --- | --- |
|  | Progression-free | Progressed |  |
| Complete blood count | 1·09 | 0·35 | NICE TA661 ^55^ |
| CT scan | 0·57 | 0·00 |  |
| Oncologist appointment | 1·09 | 0·35 |  |

Abbreviations: CT – computerised tomography; NICE – National Institute for Health and Care Excellence.

### **Table S32: Unit costs of health state resources**

| Parameter | Unit cost | Source |
| --- | --- | --- |
| Complete blood count | £6·95 | National Cost Collection 2023/24 ^52^ |
| CT scan | £233·39 |  |
| Oncologist appointment | £193·39 |  |

Abbreviations: CT – computerised tomography.

## Kidney Cancer Costs

Unit costs of kidney cancer treatment are detailed in Table S33. These costs have been calculated using pack and treatment unit inputs, including the pack cost, unit size, pack size, dose, cycle length and duration.

The proportions who receive second-line treatment are reported in Table S34. These proportions were split by cancer stage and by those who receive pembrolizumab as a first-line treatment. The stage 1 to 3 proportions were also split by those who are eligible for immunotherapy at second line.

The unit cost of treatment and the proportions receiving each treatment were used to determine the cost of treatment of proportion for stage 1 to 3 and stage 4 kidney cancer (reported in Table S34). These costs were then used along with the proportions diagnosed with stage 1 to 3 and stage 4 kidney cancer to estimate the one-off cost of treatment for progression of kidney cancer (see Table S35).

### **Table S33: Unit costs of Kidney cancer**

| **Parameter** | Unit cost | Source |
| --- | --- | --- |
| Nivolumab | £46,940·50 | Calculated using data from the British National Formulary 2025 ^39^ and NICE TA830 ^56^ |
| Axitinib | £45,113·38 |  |
| Cabozantinib | £63,137·83 |  |
| Pazopanib | £12,169·62 | Calculated using data from the eMIT ^50^ and NICE TA830 ^56^ |
| Everolimus | £1,842·22 |  |
| Sunitinib | £302·25 | Calculated using data from Grande et al ^57^ |
| Salvage surgery | £5,121·96 | Calculated using data from the National Cost Collection ^52^ |
| Radiotherapy | £625·04 |  |

Abbreviations: eMIT – the drugs and pharmaceutical electronic market information tool; NICE – National Institute for Health and Care Excellence.

### **Table S34: Proportions having second line treatment**

| **Parameters** | Stage 1 to 3 | | | | Stage 4 | |
| --- | --- | --- | --- | --- | --- | --- |
|  | With pembrolizumab | | Without pembrolizumab | | With pembrolizumab | Without pembrolizumab |
|  | IO-ineligible | IO-eligible | IO-ineligible | IO-eligible |  |  |
| Nivolumab | 15·00% | 0·00% | 15·00% | 0·00% | 0·00% | 30·00% |
| Axitinib | 7·00% | 7·00% | 5·00% | 7·00% | 0·00% | 7·50% |
| Cabozantinib | 32·00% | 32·00% | 25·00% | 32·00% | 0·00% | 12·50% |
| Pazopanib | 4·00% | 4·00% | 0·00% | 4·00% | 30·00% | 0·00% |
| Everolimus | 7·00% | 7·00% | 5·00% | 7·00% | 0·00% | 0·00% |
| Sunitinib | 0·00% | 0·00% | 0·00% | 0·00% | 20·00% | 0·00% |
| Salvage surgery | 21·50% | 21·50% | 21·50% | 21·50% | 6·61% | 6·61% |
| Radiotherapy | 0·00% | 0·00% | 0·00% | 0·00% | 13·21% | 13·21% |

### **Table S35: Progression treatment costs and proportions**

| **Parameter** | Value | Source |
| --- | --- | --- |
| Proportion diagnosed in stage 1 to 3 | 74·75% | NHS England ^53^ |
| Proportion diagnosed in stage 4 | 25·25% |  |
| Proportion treated with pembrolizumab at first line (stage 1 to 3) | 33·00% | Assumption based on NICE TA858 ^58^ |
| Proportion treated with pembrolizumab at first line (stage 4) | 33·00% |  |
| Proportion eligible for immunotherapy at second line (stage 1 to 3) | 25·00% |  |
| Cost of treatment for stage 1 to 3 | £27,422 | Calculated |
| Cost of treatment for stage 4 | £18,636 |  |
| **Total one-off cost of progression** | **£25,204** |  |

Abbreviations: NHS – National Health Service; NICE – National Institute for Health and Care Excellence.

## Health state costs

The monthly progression-free and progressed resources are reported in Table S36. The progressed stage 1 to 3 resources were split by those who have LRR and DM, and it was assumed that 50% of people are LRR.

These proportions were applied to the unit costs, presented in Table S37, and together were used to calculate the monthly cost of being progression-free and progressed, which were calculated to be £80·11 for progression-free and £176·17 for progressed. These monthly costs were then applied to those in the progression-free or progressed health states each cycle within the model.

### **Table S36: Health state resources (per month)**

| Parameter | Health state resources (Stage 1 to 3) | | | Health state resources (Stage 4) | |
| --- | --- | --- | --- | --- | --- |
|  | Progression-free | Progressed - LRR | Progressed - DM | Progression-free | Progressed |
| Complete blood count | 0·16 | 0·08 | 1·00 | 1·00 | 1·00 |
| CT scan | 0·08 | 0·08 | 0·25 | 0·25 | 0·25 |
| Oncologist appointment | 0·00 | 0·00 | 1·00 | 1·00 | 1·00 |

Abbreviations: CT – computerised tomography, DM – disease metastasis; LRR – locoregional recurrence.

### **Table S37: Unit costs of health state resources**

| Parameter | Unit cost | Source |
| --- | --- | --- |
| Complete blood count | £6·95 | National Cost Collection 2023/24 ^52^ |
| CT scan | £233·39 |  |
| Oncologist appointment | £193·39 |  |

Abbreviations: CT – computerised tomography.

## General Cancer Costs

For the general cancer population, lung cancer and head and neck cancer costs were used to estimate the one-off treatment cost of progression, and the monthly progression-free and progressed costs. Lung cancer and head and neck cancer costs were used because these cancer sites were two of the most common in Cinciripini et al. (2024),^59^ where the Kaplan-Meier (KM) data was sourced from. In the base case analysis, an average of the lung cancer and head and neck cancer costs was used, presented in Table S38.

### **Table S38: The total costs for general cancer**

| **Parameter** | Unit cost | Source |
| --- | --- | --- |
| One-off progression treatment cost | £15,234 | Calculated by averaging the lung cancer and head and neck cancer costs |
| Monthly progression free cost | £255 |  |
| Monthly progressed cost | £445 |  |

## Administration cost

The cost of delivering subsequent elements of chemotherapy via intravenous push was sourced from the National Cost Collection.^60^ The total cost of administration was calculated using the cycle length and duration of treatment information for the medications that are administered using IV. For medications that are taken by oral tablets, no cost was applied in the model.

## End of life cost

End-of-life costs were applied as a one-off cost to the modelled cohort upon entering the ‘dead’ health state. A cost of £13,409 was sourced from the Personal Social Services Research Unit, using the hospital and social care cost of dying from cancer.^36^

Health-related quality of life

Health state utilities for each of the populations are displayed in Table S39. These utilities were applied multiplicatively to the background utilities using the age and proportion male values reported in Table S40. In the base case, background utility was informed by EQ-5D data published by the NICE Decision Support Unit.^14^

The current and former smoker disutility values, presented in Table S41, were used to calculate the utility decrement from smoking.

### **Table S39: Cancer-related utility values**

| **Utilities** | Proportion-free | Progressed | Source |
| --- | --- | --- | --- |
| Lung cancer | 0·81 | 0·67 | NICE TA1037 (2025) ^61^ |
| Head and neck cancer | 0·86 | 0·28 | van der Linden (2015) ^62^ |
| Kidney cancer | 0·73 | 0·65 | NICE TA830 (2022) ^56^ |
| General cancer | 0·78 | 0·45 | Average of lung and head and neck values |

Abbreviations: NICE – National Institute for Health and Care Excellence.

### **Table S40: Demographic information to information adjustment factors**

| **Demographic values** | Age | Proportion male | Source |
| --- | --- | --- | --- |
| Lung cancer | 64 years old | 31·70% | NICE TA1037 (2025) ^61^ |
| Head and neck cancer | 57 years old | 87·36% | Hit et al., (2022) ^63^ |
| Kidney cancer | 59 years old | 72·34% | NICE TA512 (2018)^64^ |
| General cancer | 62 years old | 59·53% | Average of lung and head and neck values |

Abbreviations: NICE – National Institute for Health and Care Excellence.

### **Table S41: Smoking-related disutility values**

| **Disutilities** | Disutility | Source |
| --- | --- | --- |
| Current smoker | 0·034 | Vogl et al., 2012 ^65^ |
| Former smoker | 0·017 |  |

Results: Deterministic base case results

Tables S42 to S45 display the per-person cost breakdown for smoking cessation in each population. Table S46 displays the proportional cost-breakdown per population.

### **Table S42: Per-person cost breakdown for the lung cancer population**

| Cost breakdown (per person) | Used at diagnosis | Usual care | Incremental |
| --- | --- | --- | --- |
| Total cost of those who quit smoking | £11,275 | £3,847 | £7,427 |
| Total cost of those who continued smoking | £49,844 | £57,056 | -£7,212 |
| **Cancer treatment costs** | **£58,596** | **£58,347** | **£248** |
| Progression free | £18,470 | £17,808 | £662 |
| Progressed | £29,384 | £29,715 | -£331 |
| End of life | £10,742 | £10,824 | -£82 |
| **Comorbidity costs** | **£2,523** | **£2,556** | **-£33** |
| COPD | £165 | £166 | £0 |
| CHD | £912 | £933 | -£21 |
| Myocardial Infarction | £181 | £185 | -£4 |
| Type 2 Diabetes | £229 | £228 | £1 |
| Stroke | £654 | £667 | -£12 |
| Dementia | £381 | £378 | £3 |
| Asthma | £0 | £0 | £0 |
| **Smoking cessation treatment costs** | **£211** | **£134** | **£77** |
| Behavioural support alone | £17 | £66 | -£49 |
| Cytisine | £8 | £0 | £8 |
| Varenicline | £9 | £7 | £2 |
| Nicotine containing E-cigarettes | £22 | £35 | -£13 |
| Varenicline + NRT l/s | £0 | £0 | £0 |
| E-cigarettes + NRT l/s | £59 | £0 | £59 |
| **NRT alone costs** | **£50** | **£18** | **£32** |
| **Implementation costs** | **£46** | **£8** | **£38** |

Abbreviations: COPD – chronic obstructive pulmonary disease; CHD – coronary heart disease; NRT – nicotine replacement therapy.

### **Table S43: Per-person cost breakdown for the head and neck cancer population**

| Cost breakdown (per person) | Used at diagnosis | Usual care | Incremental |
| --- | --- | --- | --- |
| Total cost of those who quit smoking | £8,417 | £2,872 | £5,545 |
| Total cost of those who continued smoking | £31,436 | £35,984 | -£4,548 |
| **Cancer treatment costs** | **£37,860** | **£36,864** | **£996** |
| Progression free | £25,726 | £24,577 | £1,149 |
| Progressed | £2,074 | £2,081 | -£7 |
| End of life | £10,061 | £10,206 | -£146 |
| **Comorbidity costs** | **£1,993** | **£1,992** | **£1** |
| COPD | £179 | £177 | £2 |
| CHD | £686 | £693 | -£7 |
| Myocardial Infarction | £182 | £184 | -£1 |
| Type 2 Diabetes | £267 | £262 | £5 |
| Stroke | £457 | £459 | -£2 |
| Dementia | £222 | £217 | £4 |
| Asthma | £0 | £0 | £0 |
| **Smoking cessation treatment costs** | **£211** | **£134** | **£77** |
| Behavioural support alone | £17 | £66 | -£49 |
| Cytisine | £8 | £0 | £8 |
| Varenicline | £9 | £7 | £2 |
| Nicotine containing E-cigarettes | £22 | £35 | -£13 |
| Varenicline + NRT l/s | £0 | £0 | £0 |
| E-cigarettes + NRT l/s | £59 | £0 | £59 |
| **NRT alone costs** | **£50** | **£18** | **£32** |
| **Implementation costs** | **£46** | **£8** | **£38** |

Abbreviations: COPD – chronic obstructive pulmonary disease; CHD – coronary heart disease; NRT – nicotine replacement therapy.

### **Table S44: Per-person cost breakdown for the kidney cancer population**

| Cost breakdown (per person) | Used at diagnosis | Usual care | Incremental |
| --- | --- | --- | --- |
| Total cost of those who quit smoking | £6,747 | £2,302 | £4,445 |
| Total cost of those who continued smoking | £32,560 | £37,270 | -£4,711 |
| **Cancer treatment costs** | **£36,918** | **£37,228** | **-£310** |
| Progression free | £7,077 | £6,619 | £458 |
| Progressed | £20,518 | £21,047 | -£529 |
| End of life | £9,323 | £9,562 | -£239 |
| **Comorbidity costs** | **£2,388** | **£2,345** | **£44** |
| COPD | £222 | £217 | £5 |
| CHD | £820 | £815 | £5 |
| Myocardial Infarction | £225 | £224 | £1 |
| Type 2 Diabetes | £330 | £321 | £9 |
| Stroke | £542 | £534 | £9 |
| Dementia | £250 | £235 | £15 |
| Asthma | £0 | £0 | £0 |
| **Smoking cessation treatment costs** | **£211** | **£134** | **£77** |
| Behavioural support alone | £17 | £66 | -£49 |
| Cytisine | £8 | £0 | £8 |
| Varenicline | £9 | £7 | £2 |
| Nicotine containing E-cigarettes | £22 | £35 | -£13 |
| Varenicline + NRT l/s | £0 | £0 | £0 |
| E-cigarettes + NRT l/s | £59 | £0 | £59 |
| NRT alone costs | £50 | £18 | £32 |
| Implementation costs | £46 | £8 | £38 |

Abbreviations: COPD – chronic obstructive pulmonary disease; CHD – coronary heart disease; NRT – nicotine replacement therapy.

### **Table S45: Per-person cost breakdown for the general cancer population**

| Cost breakdown (per person) | Used at diagnosis | Usual care | Incremental |
| --- | --- | --- | --- |
| Total cost of those who quit smoking | £10,929 | £3,729 | £7,199 |
| Total cost of those who continued smoking | £47,577 | £54,461 | -£6,884 |
| **Cancer treatment costs** | **£55,327** | **£54,929** | **£398** |
| Progression free | £31,174 | £30,696 | £478 |
| Progressed | £14,784 | £14,803 | -£19 |
| End of life | £9,369 | £9,430 | -£61 |
| **Comorbidity costs** | **£3,179** | **£3,261** | **-£82** |
| COPD | £244 | £247 | -£3 |
| CHD | £1,104 | £1,143 | -£38 |
| Myocardial Infarction | £257 | £265 | -£8 |
| Type 2 Diabetes | £348 | £351 | -£3 |
| Stroke | £766 | £789 | -£23 |
| Dementia | £460 | £466 | -£6 |
| Asthma | £0 | £0 | £0 |
| **Smoking cessation treatment costs** | **£211** | **£134** | **£77** |
| Behavioural support alone | £17 | £66 | -£49 |
| Cytisine | £8 | £0 | £8 |
| Varenicline | £9 | £7 | £2 |
| Nicotine containing E-cigarettes | £22 | £35 | -£13 |
| Varenicline + NRT l/s | £0 | £0 | £0 |
| E-cigarettes + NRT l/s | £59 | £0 | £59 |
| NRT alone costs | £50 | £18 | £32 |
| Implementation costs | £46 | £8 | £38 |

Abbreviations: COPD – chronic obstructive pulmonary disease; CHD – coronary heart disease; NRT – nicotine replacement therapy.

### **Table S46: Costs per-person as a proportion of the total for each model arm**

| Proportional costs | Used at diagnosis | | Usual care | |
| --- | --- | --- | --- | --- |
|  | **Costs per person** | **% of total** | **Costs per person** | **% of total** |
| **Lung cancer population** | | | | |
| Total costs | £61,329 | 100·00% | £61,037 | 100·00% |
| Cancer treatment costs | £58,596 | 95·54% | £58,347 | 95·59% |
| Comorbidity costs | £2,523 | 4·11% | £2,556 | 4·19% |
| Smoking cessation costs | £211 | 0·34% | £134 | 0·22% |
| **Head and neck cancer population** | | | | |
| Total costs | £40,064 | 100·00% | £38,990 | 100·00% |
| Cancer treatment costs | £37,860 | 94·50% | £36,864 | 94·55% |
| Comorbidity costs | £1,993 | 4·97% | £1,992 | 5·11% |
| Smoking cessation costs | £211 | 0·53% | £134 | 0·34% |
| **Kidney cancer population** | | | | |
| Total costs | £39,517 | 100·00% | £39,707 | 100·00% |
| Cancer treatment costs | £36,918 | 93·42% | £37,228 | 93·76% |
| Comorbidity costs | £2,388 | 6·04% | £2,345 | 5·91% |
| Smoking cessation costs | £211 | 0·53% | £134 | 0·34% |
| **General cancer population** | | | | |
| Total costs | £58,716 | 100·00% | £58,324 | 100·00% |
| Cancer treatment costs | £55,327 | 94·23% | £54,929 | 94·18% |
| Comorbidity costs | £3,179 | 5·41% | £3,261 | 5·59% |
| Smoking cessation costs | £211 | 0·36% | £134 | 0·23% |

Results: Deterministic sensitivity analysis (DSA)

The univariate impact for a range of parameters was tested in the DSA. The base case analysis DSA results for the four populations are presented in Figures S1 to S4. The DSA results show that varying individual parameters can impact the cost-effectiveness results of the model. The results are ranked from most impactful on the incremental net monetary benefit (NMB) to least impactful.

When all parameters are varied independently, the main drivers of the incremental NMB for all four populations are the:

- Cohort starting age.
- Proportion quitting with behavioural support.
- Uptake of smoking cessation when offered at diagnosis.
- Utility value for progression-free cancer.
- Utility value for progressed cancer.

Two-way sensitivity analysis was also conducted to vary the uptake of smoking cessation when offered at diagnosis (base case = 74·84%) and the system redesign cost (base case = £45·98). This was done to determine how the interplay between these two parameters impacts the results.

The results revealed that at every point where the uptake of smoking cessation when offered at diagnosis and the system redesign cost are varied, the results are cost effective. This was consistent across all four populations.

### **Figure S1: Tornado diagram for Lung cancer**

### **Figure S2: Tornado diagram for Head and neck cancer**

### **Figure S3: Tornado diagram for Kidney cancer**

### **Figure S4: Tornado diagram for General cancer**

Results: Probabilistic sensitivity analysis (PSA)

PSA convergence was met after approximately 100 iterations. PSA results for each cancer population are presented in the following sections.

## Lung cancer

### **Table S47: PSA mean outcomes per person for the lung cancer population**

| **Outcome**  **Mean (LCI – UCI)** | **Offered at diagnosis** | **Usual care** | **Incremental** |
| --- | --- | --- | --- |
| Total cost | £61,969  (£52,784 - £71,980) | £61,553  (£52,350 - £72,278) | £416  (-£979 - £2,253) |
| Total QALYs | 5·69  (5·23 - 6·14) | 5·47  (5·03 - 5·89) | 0·22  (0·06 - 0·53) |
| Total life years | 3·22  (2·31 - 3·88) | 3·08  (2·21 - 3·69) | 0·14  (0·04 - 0·35) |
| **Incremental Cost-Effectiveness Ratio (ICER)** | | | £2,878 |
| Incremental NMB per person | | | £2,477  (£525 - £6,237) |
| Incremental NHB per person | | | 0·12  (0·03 - 0·31) |
| **Probability of cost-effectiveness** | | | 99·70% |
| **Probability of dominance (QALY increasing and cost saving)** | | | 30·00% |

Abbreviations: LCI – lower confidence interval; NHB – net health benefit; NMB – net monetary benefit; QALY – quality-adjusted life year; UCI – upper confidence interval.

## Head and neck cancer

### **Table S48: PSA mean outcomes per person for the head and neck cancer population**

| **Outcome**  **Mean (LCI – UCI)** | **Offered at diagnosis** | **Usual care** | **Incremental** |
| --- | --- | --- | --- |
| Total cost | £40,274  (£33,168 - £48,245) | £38,950  (£32,057 - £46,791) | £1,324  (£333 - £3,200) |
| Total QALYs | 6·86  (6·28 - 7·48) | 6·49  (5·97 - 7·01) | 0·37  (0·12 - 0·85) |
| Total life years | 4·17  (2·83 - 5·09) | 3·93  (2·67 - 4·75) | 0·25  (0·07 - 0·59) |
| **Incremental Cost-Effectiveness Ratio (ICER)** | | | £5,363 |
| Incremental NMB per person | | | £3,614  (£897 - £8,781) |
| Incremental NHB per person | | | 0·18  (0·04 - 0·44) |
| **Probability of cost-effectiveness** | | | 99·90% |
| **Probability of dominance (QALY increasing and cost saving)** | | | 0·20% |

Abbreviations: LCI – lower confidence interval; NHB – net health benefit; NMB – net monetary benefit; QALY – quality-adjusted life year; UCI – upper confidence interval.

## Kidney cancer

### **Table S49: PSA mean outcomes per person for the kidney cancer population**

| **Outcome**  **Mean (LCI – UCI)** | **Offered at diagnosis** | **Usual care** | **Incremental** |
| --- | --- | --- | --- |
| Total cost | £39,796  (£34,402 - £46,037) | £40,044  (£34,406 - £46,254) | -£248  (-£1,161 - £414) |
| Total QALYs | 8·56  (7·58 - 9·55) | 7·98  (7·07 - 8·92) | 0·58  (0·16 - 1·39) |
| Total life years | 4·61  (3·38 - 5·69) | 4·28  (3·11 - 5·24) | 0·33  (0·10 - 0·80) |
| **Incremental Cost-Effectiveness Ratio (ICER)** | | | Dominant |
| Incremental NMB per person | | | £6,893  (£2,234 - £16,204) |
| Incremental NHB per person | | | 0·34  (0·11 - 0·81) |
| **Probability of cost-effectiveness** | | | 100·00% |
| **Probability of dominance (QALY increasing and cost saving)** | | | 70·40% |

Abbreviations: LCI – lower confidence interval; NHB – net health benefit; NMB – net monetary benefit; QALY – quality-adjusted life year; UCI – upper confidence interval.

## General cancer

### **Table S50: PSA mean outcomes per person for the general cancer population**

| **Outcome**  **Mean (LCI – UCI)** | **Offered at diagnosis** | **Usual care** | **Incremental** |
| --- | --- | --- | --- |
| Total cost | £59,126  (£50,738 - £67,861) | £58,626  (£50,262 - £67,113) | £499  (-£114 - £1,485) |
| Total QALYs | 8·63  (8·35 - 8·94) | 8·45  (8·19 - 8·70) | 0·17  (0·06 - 0·42) |
| Total life years | 5·06  (3·47 - 6·04) | 4·93  (3·37 - 5·90) | 0·13  (0·04 - 0·33) |
| **Incremental Cost-Effectiveness Ratio (ICER)** | | | £3,845 |
| Incremental NMB per person | | | £2,098  (£270 - £5,876) |
| Incremental NHB per person | | | 0·10  (0·01 - 0·29) |
| **Probability of cost-effectiveness** | | | 97·80% |
| **Probability of dominance (QALY increasing and cost saving)** | | | 8·80% |

Abbreviations: LCI – lower confidence interval; NHB – net health benefit; NMB – net monetary benefit; QALY – quality-adjusted life year; UCI – upper confidence interval.

Results: threshold analysis

Threshold analysis was conducted to determine the lowest uptake of smoking cessation required to enable the deterministic ICER to fall just below the chosen cost-effectiveness threshold, given all other parameters in the model remain the same. The results are relative to a willingness-to-pay threshold of £20,000 per QALY gained. The threshold graphs for each population are provided:

### **Figure S5: Threshold analysis of smoking cessation uptake for lung cancer population**

### **Figure S6: Threshold analysis of smoking cessation uptake for head and neck cancer population**

### **Figure S7: Threshold analysis of smoking cessation uptake for kidney cancer population**

### **Figure S8: Threshold analysis of smoking cessation uptake for general cancer population**

Further threshold analysis was also conducted on the cost of the e-consultation to reflect the cost of implementing smoking cessation at the point of cancer diagnosis, as this value is currently unknown. The cost was varied in £100 increments to determine the cost at which point implementing smoking cessation services are no longer cost effective relative to a £20,000 per QALY gained threshold. The threshold graphs for each population are provided:

### **Figure S9: Threshold analysis of e-consultation cost for lung cancer population**

### **Figure S10: Threshold analysis of e-consultation cost for head and neck cancer population**

### **Figure S11: Threshold analysis of e-consultation cost for kidney cancer population**

### **Figure S12: Threshold analysis of e-consultation cost for general cancer population**

Results: scenario analysis

## Scenario 1: Using different parametric models

The model includes functionality to select one of six parametric models to fit to the survival data. These parametric models are exponential, Weibull, Gompertz, log-normal, log-logistic, and generalised gamma. In the base case, the distributions were selected based on best statistic fit (using goodness-of-fit measures), visual fit and clinical plausibility. Scenario analysis was conducted whereby alternative distributions were used to estimate the long-term extrapolations.

The scenario analysis indicated that changing the selected distribution to any of the other five parametric models, that are not used in the base case, had no substantial impact on the model outcomes for any of the four cancer populations.

## Scenario 2: Using the kidney cancer KM data to calculate the hazard ratios for PFS

Hazard ratios were calculated between overall survival (OS) and progression-free survival (PFS) using pseudo-independent patient data. In the base case, these hazard ratios were generated using the lung cancer KM data. In this scenario, the hazard ratios have been calculated using the kidney cancer KM data instead, to determine the impact on the model outcomes. Table S51 displays the hazard ratios calculated using lung cancer KM data and kidney cancer KM data.

Table S52 shows the base case and scenario analysis results for both the head and neck cancer and general cancer populations (where PFS KM data could not be sourced). The results indicate that when hazard ratios from Kidney cancer were used instead of lung cancer hazard ratios, there was minimal change in results for the two populations that used hazard ratios for OS versus PFS in their base case (head and neck cancer, and general cancer).

### **Table S51: Hazard ratios for quit and continued smoking for OS versus PFS**

| **Parameter** | Lung cancer KM (base case) | Kidney cancer KM (scenario 2) |
| --- | --- | --- |
| Quit smoking hazard ratio | 0·87 | 0·79 |
| Continued smoking hazard ratio | 0·89 | 0·80 |

Abbreviations: KM – Kaplan-Meier.

### **Table S52: Per-person summary results for scenario 2 as compared to the base case**

| **Per person results** | | Population | |
| --- | --- | --- | --- |
|  |  | Head and neck cancer | General cancer |
| **Base case** | Incremental cost | £1,074 | £392 |
|  | Incremental LY | 0·29 | 0·13 |
|  | Incremental QALY | 0·20 | 0·10 |
|  | **ICER** | **£5,495** | **£4,055** |
|  | NMB | £2,834 | £1,543 |
|  | NHB | 0·14 | 0·08 |
| **Scenario 2** | Incremental cost | £1,083 | £367 |
|  | Incremental LY | 0·29 | 0·13 |
|  | Incremental QALY | 0·20 | 0·10 |
|  | **ICER** | **£5,501** | **£3,698** |
|  | NMB | £2,855 | £1,618 |
|  | NHB | 0·14 | 0·08 |

Abbreviations: ICER – incremental cost-effectiveness ratio; LY – life year; NHB – net health benefit; NMB – net monetary benefit; QALY – quality-adjusted life year.

## Scenario 3: Time horizon

The per person results across time horizons of 1 year, 5 years, 10 years, 15 years, and lifetime (base case) are presented in Table S53. Across all populations, implementing smoking cessation into cancer care is cost-saving and quality-adjusted life year (QALY)-increasing at the 1-year time horizon. Kidney cancer is the only population where the incremental cost-effectiveness ratio (ICER) is dominant across all time horizons.

For the lung cancer population, the ICER is dominant if a time horizon of 13 years or lower is selected. For a time horizon of 14 years and upwards, smoking cessation begins to incur costs. However, health benefits are observed across all time horizons and smoking cessation is cost-effective at the willingness-to-pay threshold of £20,000 per QALY gained across all time horizons.

For the head and neck cancer population, the ICER is dominant when a time horizon of three years or lower is used. Similarly, for the general cancer population a time horizon of seven years or lower results in a dominant ICER. For both of these populations, health benefits are observed across every time horizon, and the redesign of smoking cessation services is cost-effective at the willingness-to-pay threshold of £20,000 per QALY gained across all time horizons.

### **Table S53: Results across different time horizons**

| **Time horizon** | **Per person results** | Population | | | |
| --- | --- | --- | --- | --- | --- |
|  |  | Lung cancer | Head and neck cancer | Kidney cancer | General cancer |
| **1 year** | Incremental cost | -£124 | -£124 | -£158 | -£128 |
|  | Incremental QALY | 0·00 | 0·01 | 0·00 | 0·00 |
|  | **ICER** | **Dominant** | **Dominant** | **Dominant** | **Dominant** |
| **5 years** | Incremental cost | -£350 | £99 | -£760 | -£89 |
|  | Incremental QALY | 0·03 | 0·06 | 0·06 | 0·04 |
|  | **ICER** | **Dominant** | **£1,626** | **Dominant** | **Dominant** |
| **10 years** | Incremental cost | -£157 | £431 | -£940 | £94 |
|  | Incremental QALY | 0·7 | 0·11 | 0·16 | 0·08 |
|  | **ICER** | **Dominant** | **£3,810** | **Dominant** | **£1,471** |
| **15 years** | Incremental cost | £38 | £692 | -£847 | £226 |
|  | Incremental QALY | 0·10 | 0·15 | 0·28 | 0·11 |
|  | **ICER** | **£388** | **£4,554** | **Dominant** | **£2,742** |
| **Lifetime (base case)** | Incremental cost | £292 | £1,074 | -£189 | £392 |
|  | Incremental QALY | 0·11 | 0·20 | 0·45 | 0·13 |
|  | **ICER** | **£2,606** | **£5,495** | **Dominant** | **£4,055** |

Abbreviations: ICER – incremental cost-effectiveness ratio; QALY – quality-adjusted life year.

## Scenario 4: Benefits of smoking cessation for cancer outcomes

A scenario was conducted to determine the standalone benefits of smoking cessation for cancer outcomes. Hence, the additional benefits from smoking-related illnesses were excluded. This scenario allows the model to isolate the impact of smoking cessation, which will reflect an analysis that determines the exact impact that smoking cessation has on cancer outcomes.

To conduct this scenario the costs and disutilities for the comorbidities were set the zero. Table S54 displays the summary results for the scenario analysis and the base case for the four populations. The scenario analysis results are consistent to the base case results, indicating that the cancer-related outcomes drive the model results.

### **Table S54: Per-person summary results for scenario 4 as compared to the base case**

| **Per person results** | | Population | | | |
| --- | --- | --- | --- | --- | --- |
|  |  | Lung cancer | Head and neck cancer | Kidney cancer | General cancer |
| **Base case** | Incremental cost | £292 | £1,074 | -£189 | £392 |
|  | Incremental LY | 0·17 | 0·29 | 0·45 | 0·13 |
|  | Incremental QALY | 0·11 | 0·20 | 0·26 | 0·10 |
|  | **ICER** | **£2,606** | **£5,495** | **Dominant** | **£4,055** |
|  | NMB | £1,951 | £2,834 | £5,362 | £1,543 |
|  | NHB | 0·10 | 0·14 | 0·27 | 0·08 |
| **Scenario analysis: cancer outcomes alone** | Incremental cost | £325 | £1,073 | -£233 | £475 |
|  | Incremental LY | 0·17 | 0·29 | 0·45 | 0·13 |
|  | Incremental QALY | 0·11 | 0·20 | 0·26 | 0·10 |
|  | **ICER** | **£2,916** | **£5,444** | **Dominant** | **£4,939** |
|  | NMB | £1,905 | £2,868 | £5,467 | £1,448 |
|  | NHB | 0·10 | 0·14 | 0·27 | 0·07 |

Abbreviations: ICER – incremental cost-effectiveness ratio; LY – life year; NHB – net health benefit; NMB – net monetary benefit; QALY – quality-adjusted life year.

## Scenario 5: Benefits of smoking cessation for smoking-related illnesses

This scenario was conducted to determine the standalone benefits of smoking cessation for smoking-related illnesses. To conduct this scenario costs and utility values associated with progression-free cancer, progressed cancer and end-of-life were set to zero in the model. Table S55 displays the summary results for the four populations for scenario 5 as compared to the base case.

### **Table S55: Per-person summary results for scenario 5 as compared to the base case**

| **Per person results** | | Population | | | |
| --- | --- | --- | --- | --- | --- |
|  |  | Lung cancer | Head and neck cancer | Kidney cancer | General cancer |
| **Base case** | Incremental cost | £292 | £1,074 | -£189 | £392 |
|  | Incremental LY | 0·17 | 0·29 | 0·45 | 0·13 |
|  | Incremental QALY | 0·11 | 0·20 | 0·26 | 0·10 |
|  | **ICER** | **£2,606** | **£5,495** | **Dominant** | **£4,055** |
|  | NMB | £1,951 | £2,834 | £5,362 | £1,543 |
|  | NHB | 0·10 | 0·14 | 0·27 | 0·08 |
| **Scenario analysis: smoking-related illness outcomes alone** | Incremental cost | £44 | £77 | £120 | -£6 |
|  | Incremental LY | 0·17 | 0·29 | 0·45 | 0·13 |
|  | Incremental QALY | 0·01 | 0·01 | 0·01 | 0·02 |
|  | **ICER** | **£5,009** | **£12,137** | **£23,697** | **Dominant** |
|  | NMB | £131 | £50 | -£19 | £317 |
|  | NHB | 0·01 | 0·00 | 0·00 | 0·02 |

Abbreviations: ICER – incremental cost-effectiveness ratio; LY – life year; NHB – net health benefit; NMB – net monetary benefit; QALY – quality-adjusted life year.

## Scenario 6: Equal treatments given across pathways

This scenario was conducted to determine how much the impact of different smoking cessation methods has on the results of the model. In this scenario smoking cessation interventions are set equal regardless of whether services are provided as part of cancer treatment, or through self-referral methods to the centralised hub. Table S56 displays the summary results for the four populations for scenario 6 as compared to the base case. The results suggest that regardless of the smoking cessation method given, redesigning smoking cessation services is likely to be cost-effective, with minimal impact on the overall results.

### **Table S56: Per-person summary results for scenario 6 as compared to the base case**

| **Per person results** | | Population | | | |
| --- | --- | --- | --- | --- | --- |
|  |  | Lung cancer | Head and neck cancer | Kidney cancer | General cancer |
| **Base case** | Incremental cost | £292 | £1,074 | -£189 | £392 |
|  | Incremental LY | 0·17 | 0·29 | 0·45 | 0·13 |
|  | Incremental QALY | 0·11 | 0·20 | 0·26 | 0·10 |
|  | **ICER** | **£2,606** | **£5,495** | **Dominant** | **£4,055** |
|  | NMB | £1,951 | £2,834 | £5,362 | £1,543 |
|  | NHB | 0·10 | 0·14 | 0·27 | 0·08 |
| **Scenario analysis: smoking-related illness outcomes alone** | Incremental cost | £229 | £924 | -£198 | £318 |
|  | Incremental LY | 0·15 | 0·26 | 0·40 | 0·11 |
|  | Incremental QALY | 0·10 | 0·17 | 0·23 | 0·09 |
|  | **ICER** | **£2,303** | **£5,321** | **Dominant** | **£3,703** |
|  | NMB | £1,764 | £2,548 | £4,794 | £1,401 |
|  | NHB | 0·09 | 0·13 | 0·24 | 0·07 |

Abbreviations: ICER – incremental cost-effectiveness ratio; LY – life year; NHB – net health benefit; NMB – net monetary benefit; QALY – quality-adjusted life year.

## Scenario 7: Smoking cessation intervention

This scenario was used to understand the impact that pharmacological interventions have on economic outcomes when used as smoking cessation for people diagnosed with cancer who currently smoke. In this scenario pharmacological interventions (varenicline and cytisine) were compared against behavioural support alone. Therefore, we are not only considering where to offer people smoking cessation, but also about the type of smoking cessation offered.

In one scenario, all individuals in both the intervention and comparator arms were offered behavioural support alone (scenario 7a). This was compared to an alternative scenario (scenario 7b) in which, at the time of cancer diagnosis, 50% of individuals in the intervention arm were offered varenicline and the remaining 50% were offered cytisine, while those in the comparator arm continued to receive behavioural support only.

Table S57 displays the results of this scenario. The ICER values have not been reported as these values can be easily misinterpreted in certain scenarios. In this instance, the ICER values are slightly higher for scenario 7b because more money is spent for less health. However, more money is being spent cost-effectively compared to the rest of the system, meaning the NMB and net health benefit (NHB) are also higher, suggesting the switch to pharmacological treatments is cost-effective. Hence, the ICER values are not a valuable economic outcome in this scenario.

The results suggest that using pharmacological interventions to help people quit smoking is cost-effective than using behavioural support only. The use of varenicline and cytisine result in greater health benefits and provide a higher net value in monetary terms (as demonstrated by the NMB) across all four populations. The incremental NMB across all populations is over £1,000, and in the case of kidney cancer, is close to £4,000. This highlights the importance of also choosing the optimal smoking cessation intervention, alongside how best it is delivered or offered, to encourage the higher uptake.

### **Table S57: Per-person summary results for scenario 7 as compared to used 100% behavioural support**

| **Per person results** | | Population | | | |
| --- | --- | --- | --- | --- | --- |
|  |  | Lung cancer | Head and neck cancer | Kidney cancer | General cancer |
| **Scenario 7a:**  **100% behavioural support in both arms** | Incremental cost | £96 | £306 | -£34 | £123 |
|  | Incremental LY | 0·05 | 0·08 | 0·12 | 0·03 |
|  | Incremental QALY | 0·03 | 0·05 | 0·07 | 0·03 |
|  | NMB | £508 | £745 | £1,426 | £398 |
|  | NHB | 0·03 | 0·04 | 0·07 | 0·02 |
| **Scenario 7b:**  **Intervention arm offered pharmacological interventions and comparator arm offered behavioural support** | Incremental cost | £379 | £1,154 | -£99 | £478 |
|  | Incremental LY | 0·17 | 0·28 | 0·44 | 0·13 |
|  | Incremental QALY | 0·11 | 0·19 | 0·26 | 0·10 |
|  | NMB | £1,846 | £2,722 | £5,230 | £1,441 |
|  | NHB | 0·09 | 0·14 | 0·26 | 0·07 |

Abbreviations: ICER – incremental cost-effectiveness ratio; LY – life year; NHB – net health benefit; NMB – net monetary benefit; QALY – quality-adjusted life year.

## Scenario 8: Smoking cessation intervention

This scenario was used to understand the impact of including an odds ratio reported by Gummerson et al. (2023), that indicated that those with people were 3.23x more likely to quit smoking at 1-year after lung cancer diagnosis. This odds ratio was applied to the estimated quit rates in each arm, which were 18.01% when smoking cessation was offered at diagnosis, and 6.15% for usual care. The results displayed in Table S58 indicate that there is minimal impact on the ICER, albeit that both increment costs and QALYs increase in Scenario 8.

### **Table S58: Per-person summary results for scenario 8 as compared to the base-case lung cancer population results**

| **Per person results** | | Lung cancer |
| --- | --- | --- |
| **Base case** | Incremental cost | £292 |
|  | Incremental LY | 0·17 |
|  | Incremental QALY | 0·11 |
|  | **ICER** | **£2,606** |
|  | NMB | £1,951 |
|  | NHB | 0·10 |
| **Scenario 8 (3.23 odds ratio applied to quit rates in both arms)** | Incremental cost | £773 |
|  | Incremental LY | 0.55 |
|  | Incremental QALY | 0.36 |
|  | **ICER** | **£2,133** |
|  | NMB | £6,473 |
|  | NHB | 0.32 |

Abbreviations: ICER – incremental cost-effectiveness ratio; LY – life year; NHB – net health benefit; NMB – net monetary benefit; QALY – quality-adjusted life year.

References

1.	Audit NLC. State of the Nation Report 2024, 2024.

2.	Miquel Dominguez A, Tan EH, Burn E, et al. Incidence, prevalence, and survival of head and neck cancers in the United Kingdom from 2000-2021. *MedRxiv* 2024: 2024.12. 05.24318538.

3.	Kidney Reseach UK. What is kidney cancer? . 2025. <https://www.kidneyresearchuk.org/conditions-symptoms/kidney-cancer/#:~:text=It%20can%20come%20in%20several,age%20at%20diagnosis%20is%2064>.

4.	Cancer Research UK. Kidney cancer incidence statistics 2025. <https://www.cancerresearchuk.org/health-professional/cancer-statistics/statistics-by-cancer-type/kidney-cancer/incidence#heading-Zero>.

5.	Murray RL, Alexandris P, Baldwin D, et al. Uptake and 4-week quit rates from an opt-out co-located smoking cessation service delivered alongside community-based low-dose computed tomography screening within the Yorkshire Lung Screening Trial. *European Respiratory Journal* 2024; **63**(4).

6.	Williams PJ, Philip KE, Alghamdi SM, et al. Strategies to deliver smoking cessation interventions during targeted lung health screening - a systematic review and meta-analysis. *Chron Respir Dis* 2023; **20**: 14799731231183446.

7.	Livingstone‐Banks J, Ordóñez‐Mena JM, Hartmann‐Boyce J. Print‐based self‐help interventions for smoking cessation. *Cochrane Database of Systematic Reviews* 2019; (1).

8.	National Institute for Health and Care Excellence (NICE). Tobacco: preventing uptake, promoting quitting and treating dependence - update (NG209), 2021.

9.	Lindson N, Theodoulou A, Ordóñez-Mena JM, et al. Pharmacological and electronic cigarette interventions for smoking cessation in adults: component network meta‐analyses. *Cochrane Database of Systematic Reviews* 2023; (9).

10.	Hawkins J, Hollingworth W, Campbell R. Long-term smoking relapse: a study using the british household panel survey. *Nicotine & tobacco research* 2010; **12**(12): 1228–35.

11.	Royal College of Physicians. Hiding in plain sight: Treating tobacco dependency in the NHS 2018.

12.	Prescott E, Hippe M, Schnohr P, Hein HO, Vestbo J. Smoking and risk of myocardial infarction in women and men: longitudinal population study. *Bmj* 1998; **316**(7137): 1043.

13.	Punekar YS, Shukla A, Müllerova H. COPD management costs according to the frequency of COPD exacerbations in UK primary care. *International journal of chronic obstructive pulmonary disease* 2014: 65–73.

14.	Hernández Alava MP, S Wailoo, A. Estimating EQ-ED by age and sex for the UK 2022.

15.	Nolan CM, Longworth L, Lord J, et al. The EQ-5D-5L health status questionnaire in COPD: validity, responsiveness and minimum important difference. *Thorax* 2016; **71**(6): 493–500.

16.	Landeiro F, Harris C, Groves D, et al. The economic burden of cancer, coronary heart disease, dementia, and stroke in England in 2018, with projection to 2050: an evaluation of two cohort studies. *The Lancet Healthy Longevity* 2024; **5**(8): e514–e23.

17.	Goldsmith KA, Dyer MT, Buxton MJ, Sharples LD. Mapping of the EQ-5D index from clinical outcome measures and demographic variables in patients with coronary heart disease. *Health and quality of life outcomes* 2010; **8**(1): 54.

18.	Danese MD, Gleeson M, Kutikova L, et al. Estimating the economic burden of cardiovascular events in patients receiving lipid-modifying therapy in the UK. *BMJ open* 2016; **6**(8): e011805.

19.	Munyombwe T, Dondo T, Aktaa S, et al. Association of multimorbidity and changes in health-related quality of life following myocardial infarction: a UK multicentre longitudinal patient-reported outcomes study. *BMC medicine* 2021; **19**(1): 227.

20.	NHS England. Prescribing for Diabetes in England 2019.

21.	Stedman M, Lunt M, Davies M, et al. Cost of hospital treatment of type 1 diabetes (T1DM) and type 2 diabetes (T2DM) compared to the non-diabetes population: a detailed economic evaluation. *BMJ open* 2020; **10**(5): e033231.

22.	Boye KS, Matza LS, Stewart KD, et al. Health state utilities associated with weight loss in type 2 diabetes and obesity. *Journal of Medical Economics* 2022; **25**(1): 14–25.

23.	Xu X-M, Vestesson E, Paley L, et al. The economic burden of stroke care in England, Wales and Northern Ireland: using a national stroke register to estimate and report patient-level health economic outcomes in stroke. *European stroke journal* 2018; **3**(1): 82–91.

24.	Luengo-Fernandez R, Gray AM, Bull L, et al. Quality of life after TIA and stroke: ten-year results of the Oxford Vascular Study. *Neurology* 2013; **81**(18): 1588–95.

25.	Alzheimer's Society. Dementia UK Update 2014.

26.	Rombach I, Iftikhar M, Jhuti GS, et al. Obtaining EQ-5D-5L utilities from the disease specific quality of life Alzheimer’s disease scale: development and results from a mapping study. *Quality of Life Research* 2021; **30**(3): 867–79.

27.	Leaviss J, Sullivan W, Ren S, et al. What is the clinical effectiveness and cost-effectiveness of cytisine compared with varenicline for smoking cessation? A systematic review and economic evaluation. *Health technology assessment (Winchester, England)* 2014; **18**(33): 1.

28.	Sullivan PW, Ghushchyan VH, Campbell JD, Globe G, Bender B, Magid DJ. Measurement of utility in asthma: evidence indicating that generic instruments may miss clinically important changes. *Quality of Life Research* 2016; **25**(12): 3017–26.

29.	NHS England. Health Survey for England 2022 Part 1: Data tables, 2024.

30.	Action on Smoking and Health (ASH). Young People and Smoking 2025

31.	Stone PW, Osen M, Ellis A, Coaker R, Quint JK. Prevalence of chronic obstructive pulmonary disease in England from 2000 to 2019. *International Journal of Chronic Obstructive Pulmonary Disease* 2023: 1565–74.

32.	British Heart Foundation. Heart and Circulatory Disease Statistics 2024 2024.

33.	NHS England. Health Survey for England 2017 [NS], 2018.

34.	NHS England. Health Survey for England 2019 [NS], 2020.

35.	Bhatnagar P, Wickramasinghe K, Williams J, Rayner M, Townsend N. The epidemiology of cardiovascular disease in the UK 2014. *Heart* 2015; **101**(15): 1182–9.

36.	Jones K, Weatherly, H, Birch, S, Castelli, A, Chalkley, M, Dargan, A, Findlay, D, Gao, M, Hinde, S, Markham, S. Unit Costs of Health and Social Care 2024 Manual. 2025.

37.	NHS England. Using e-cigarettes to stop smoking 2025. <https://www.nhs.uk/live-well/quit-smoking/using-e-cigarettes-to-stop-smoking/#:~:text=E%2Dcigarettes%20are%20not%20currently,visit%20the%20Better%20Health%20website>.

38.	Li J, Hajek P, Pesola F, et al. Cost‐effectiveness of e‐cigarettes compared with nicotine replacement therapy in stop smoking services in England (TEC study): a randomized controlled trial. *Addiction* 2020; **115**(3): 507–17.

39.	National Institute for Health and Care Excellence (NICE). British National Formulary (BNF). 2025. <https://bnf.nice.org.uk/>.

40.	NHS Business Services Authority. Prescription Cost Analysis - England 2023/24, 2024.

41.	Schnoll RA, Martinez E, Tatum KL, et al. Increased self-efficacy to quit and perceived control over withdrawal symptoms predict smoking cessation following nicotine dependence treatment. *Addictive behaviors* 2011; **36**(1-2): 144–7.

42.	Nicorette. NICORETTE QuickMist - Nicotine Replacement Therapy (NRT): Consumer Medicine Information 2013.

43.	Office for National Statistics. Adult smoking habits in the UK: average cigarette consumption methodology 2024.

44.	NHS South London and Maudsley. Nicotine Mouth spray: User Guide 2020.

45.	Nicorette. NICORETTE® Inhalator 15mg Product Information. 2023.

46.	UK ECIG Store. UK ECIG Store The One Kit E-Cigarette Kit 2025. <https://www.ukecigstore.com/products/the-one-kit-by-uk-ecig-store>.

47.	Store UE. UK ECIG Store E Liquid 2025. <https://www.ukecigstore.com/search?options%5Bprefix%5D=last&page=3&q=e+liquid>.

48.	Hajek P, Phillips-Waller A, Przulj D, et al. E-cigarettes compared with nicotine replacement therapy within the UK Stop Smoking Services: the TEC RCT. *Health Technology Assessment (Winchester, England)* 2019; **23**(43): 1.

49.	National Institute for Health and Care Excellence. Durvalumab for maintenance treatment of unresectable non-small-cell lung cancer after platinum-based chemoradiation: Technology appraisal guidance [TA798]. 2022. <https://www.nice.org.uk/guidance/ta798/documents/committee-papers-2>.

50.	GOV.UK. Drugs and pharmaceutical electronic market information tool (eMIT). July 2025 2011. <https://www.gov.uk/government/publications/drugs-and-pharmaceutical-electronic-market-information-emit>.

51.	National Institite for Health and Care Ecellence. Osimertinib for adjuvant treatment of EGFR mutation-positive non-small-cell lung cancer after complete tumour resection. Technology appraisal guidance [TA1043]. 2025. <https://www.nice.org.uk/guidance/ta1043>.

52.	NHS England. National Cost Collection for the NHS. August 2025 2023. <https://www.england.nhs.uk/costing-in-the-nhs/national-cost-collection/>.

53.	NHS England. The Get Data Out programme from the National Disease Registration Service (NDRS). R Shiny App. <https://nhsd-ndrs.shinyapps.io/get_data_out/> (accessed April 2025).

54.	National Institute for Health and Care Excellence. Nivolumab with chemotherapy for neoadjuvant treatment of resectable non-small-cell lung cancer. Technology appraisal guidance [TA876]. 2023. <https://www.nice.org.uk/guidance/ta876>.

55.	National Institute for Health and Care Excellence. Pembrolizumab for untreated metastatic or unresectable recurrent head and neck squamous cell carcinoma: Technology appraisal guidance [TA661]. 2020. <https://www.nice.org.uk/guidance/ta661/history>.

56.	National Institute for Health and Care Excellence. Pembrolizumab for adjuvant treatment of renal cell carcinoma. Technology appraisal guidance [TA830]. 2022. <https://www.nice.org.uk/guidance/ta830/history>.

57.	Grande. Results from the INMUNOSUN-SOGUG trial: a prospective phase II study of sunitinib as a second-line therapy in patients with metastatic renal cell carcinoma after immune checkpoint-based combination therapy. . *ESMO Open* 2022.

58.	National Institute for Health and Care Excellence. Lenvatinib with pembrolizumab for untreated advanced renal cell carcinoma. Technology appraisal guidance [TA858]. 2023. <https://www.nice.org.uk/guidance/ta858>.

59.	Cinciripini PM, Kypriotakis G, Blalock JA, et al. Survival outcomes of an early intervention smoking cessation treatment after a cancer diagnosis. *JAMA oncology* 2024; **10**(12): 1689–96.

60.	England. N. National Cost Collection for the NHS. National schedule of NHS costs 23-24. . 2024. <https://www.england.nhs.uk/costing-in-the-nhs/national-cost-collection/> (accessed June 2025.

61.	National Instititue for Health and Care Excellence. Pembrolizumab for adjuvant treatment of resected non-small-cell lung cancer (TA1037), 2025.

62.	van der Linden N, van Gils C, Pescott C, Buter J, Vergeer M, Groot CU-d. Real-world cost-effectiveness of cetuximab in locally advanced squamous cell carcinoma of the head and neck. *European Archives of Oto-Rhino-Laryngology* 2015; **272**(8): 2007–16.

63.	Hitt R, Mesia R, Lozano A, et al. Randomized phase 3 noninferiority trial of radiotherapy and cisplatin vs radiotherapy and cetuximab after docetaxel-cisplatin-fluorouracil induction chemotherapy in patients with locally advanced unresectable head and neck cancer. *Oral Oncology* 2022; **134**: 106087.

64.	National Institute for Health and Care Excellence. Tivozanib for treating advanced renal cell carcinoma (TA512), 2018.

65.	Vogl M, Wenig CM, Leidl R, Pokhrel S. Smoking and health-related quality of life in English general population: implications for economic evaluations. *BMC public health* 2012; **12**(1): 203.

66. Gummerson SP, Lowe JT, Taylor KL, Lobo T, Jensen RE. The characteristics of patients who quit smoking in the year following a cancer diagnosis. J Cancer Surviv. 2022 Feb;16(1):111-118. doi: 10.1007/s11764-021-01009-7. Epub 2021 Feb 27. PMID: 33641030; PMCID: PMC10117081.
